# Marine-Derived 2-Aminoimidazolone Alkaloids. Leucettamine B-Related Polyandrocarpamines Inhibit Mammalian and Protozoan DYRK & CLK Kinases

**DOI:** 10.3390/md15100316

**Published:** 2017-10-17

**Authors:** Nadège Loaëc, Eletta Attanasio, Benoît Villiers, Emilie Durieu, Tania Tahtouh, Morgane Cam, Rohan A. Davis, Aline Alencar, Mélanie Roué, Marie-Lise Bourguet-Kondracki, Peter Proksch, Emmanuelle Limanton, Solène Guiheneuf, François Carreaux, Jean-Pierre Bazureau, Michelle Klautau, Laurent Meijer

**Affiliations:** 1 ManRos Therapeutics, Perharidy Research Center, 29680 Roscoff, Bretagne, France; nadege.loaec@univ-brest.fr (N.L.); attanasio@manros-therapeutics.com (E.A.); bvilliers@yahoo.fr (B.V.); durieu@manros-therapeutics.com (E.D.); tania.tahtouh@gmail.com (T.T.); cam.morgane@live.fr (M.C.); 2 Station Biologique de Roscoff, CNRS, ‘Protein Phosphorylation and Human Disease’ Group, Place G. Teissier, 29680 Roscoff, Bretagne, France; 3 Griffith Institute for Drug Discovery, Griffith University, Brisbane, QLD 4111, Australia; r.davis@griffith.edu.au; 4 Universidade Federal do Rio de Janeiro, Instituto de Biologia—Departamento de Zoologia, Av. Carlos Chagas Filho 373-CCS-Bloco A-Sala A0-100, Ilha do Fundão, 21941-902 Rio de Janeiro, Brazil; aalencar@biologia.ufrj.br; 5 Molécules de Communication et Adaptation des Micro-Organismes, UMR 7245 CNRS, Muséum National d’ Histoire Naturelle, 57 rue Cuvier (C.P. 54), 75005 Paris, France; mroue@mnhn.fr (M.R.); bourguet@mnhn.fr (M.-L.B.-K.); 6 Institut für Pharmazeutische Biologie und Biotechnologie, Universitätsstr. 1, 40225 Düsseldorf, Germany; proksch@duesseldorf-uni.de; 7 Université de Rennes 1, Institut des Sciences Chimiques de Rennes, ISCR UMR CNRS 6226, Groupe Chimie Organique et Interfaces (CORINT), Bât. 10A, Campus de Beaulieu, Avenue du Général Leclerc, CS 74205, 35042 Rennes CEDEX, Bretagne, France; emmanuelle.limanton@univ-rennes1.fr (E.L.); solene.guiheneuf@univ-rennes1.fr (S.G.)

**Keywords:** marine sponge, Porifera, Calcarea, ascidian, *Polyandrocarpa*, 2-aminoimidazolone alkaloids, leucettamine B, leucettine, polyandrocarpamines, protein kinases, DYRK, CLK, kinase inhibitor, Alzheimer’s disease, Down syndrome

## Abstract

A large diversity of 2-aminoimidazolone alkaloids is produced by various marine invertebrates, especially by the marine Calcareous sponges *Leucetta* and *Clathrina*. The phylogeny of these sponges and the wide scope of 2-aminoimidazolone alkaloids they produce are reviewed in this article. The origin (invertebrate cells, associated microorganisms, or filtered plankton), physiological functions, and natural molecular targets of these alkaloids are largely unknown. Following the identification of leucettamine B as an inhibitor of selected protein kinases, we synthesized a family of analogues, collectively named leucettines, as potent inhibitors of DYRKs (dual-specificity, tyrosine phosphorylation regulated kinases) and CLKs (cdc2-like kinases) and potential pharmacological leads for the treatment of several diseases, including Alzheimer’s disease and Down syndrome. We assembled a small library of marine sponge- and ascidian-derived 2-aminoimidazolone alkaloids, along with several synthetic analogues, and tested them on a panel of mammalian and protozoan kinases. Polyandrocarpamines A and B were found to be potent and selective inhibitors of DYRKs and CLKs. They inhibited cyclin D1 phosphorylation on a DYRK1A phosphosite in cultured cells. 2-Aminoimidazolones thus represent a promising chemical scaffold for the design of potential therapeutic drug candidates acting as specific inhibitors of disease-relevant kinases, and possibly other disease-relevant targets.

## 1. Introduction

Protein kinases catalyze the phosphorylation of proteins on serine, threonine and tyrosine residues [1]. They represent a large family of intracellular regulators which, after their initial discovery by Edwin Krebs and Edmund Fisher in the 1950s [2], has seen massive research investment on their functions and regulations. The human kinome comprises 518 kinases, many of which display some abnormal activity or deregulation in human disease. This prompted both academic and pharmaceutical laboratories to search for, optimize, characterize and develop pharmacological inhibitors of selected disease-relevant protein kinases. As a matter of fact, kinases have become the leading therapeutic target in the pharmaceutical industry, before G-protein coupled receptors, and over 30 inhibitors have already reached the clinical market [1,3,4]. Although many inhibitors were initially derived from natural products, all drug candidates are chemically synthesized at some stage of their development towards a therapeutic drug.

During a general screen for inhibitors of cell signaling and cell cycle control kinases, we identified the marine sponge product leucettamine B as a weak inhibitor of various protein kinases [5,6]. Following this initial discovery, a large number of analogues and derivatives were synthesized and optimized as kinase inhibitors, especially as inhibitors of the DYRKs (dual-specificity, tyrosine regulated kinases) and CLKs (cdc2-like kinases) kinase families. Leucettamine B-derived inhibitors are collectively named leucettines [6,7]. These inhibitors have a potential as drug candidates for the treatment of cognitive disorders associated with Down syndrome (DS) and Alzheimer’s disease (AD), but also for some cancers, inflammation, diabetes, and so forth.

**Figure 1:**
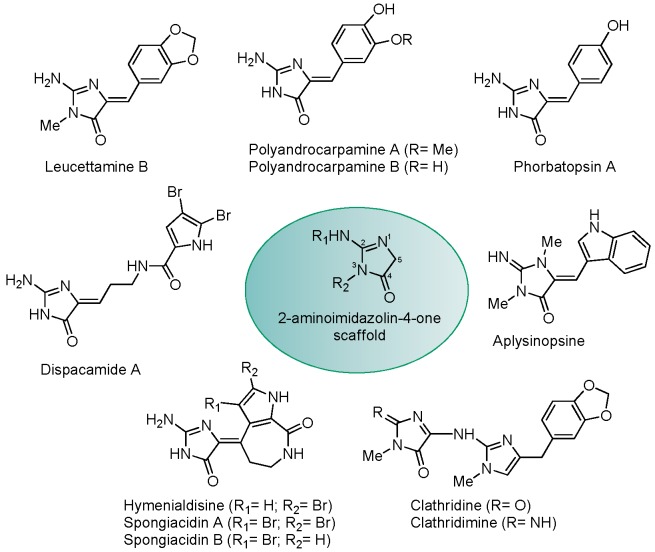
Structures of a selection of marine natural products sharing the 2-aminoimidazolin-4-one scaffold.

Leucettamine B was initially identified in the marine, calcareous sponge *Leucetta microraphis* [8]. The physiological function of this marine alkaloid is unknown, as is the biosynthetic pathway leading its production. Furthermore, whether leucettamine B is produced by the sponge itself or by some associated microorganisms remains to be determined. Interestingly, leucettamine B shows a central 2-aminoimidazolone scaffold shared by several marine products produced by various marine sponges, most of which belonging to the class Calcarea, and by other marine invertebrates, such as ascidians and nudibranchs (Figure 1). Several extensive reviews on these marine alkaloids [9,10,11] and 2-aminoimidazolones in general [12] have been recently published. We thought it might be interesting to obtain some of these leucettamine B-related natural products to evaluate them as potential kinase inhibitors. This was also an opportunity to review the sponge species producing such molecules, with particular emphasis on *Leucetta* and *Clathrina* species, as well as the wide scope of 2-aminoimidazolones produced by marine organisms.

## 2. Results

### 2.1. Phylogeny of Calcareous Sponges with Special Emphasis on Leucetta and Clathrina

Since most of the compounds described here are produced by marine calcareous sponges, we first present a phylogeny of these 2-aminoimidazolones-producing sponges, with specific focus on the *Leucetta* and *Clathrina* species. Sponges (phylum Porifera) are sessile, filter-feeding animals that utilize flagellate cells (choanocytes) to pump water through their bodies. They are morphologically very simple, as they do not have, for example, organs, sensorial cells or a nervous system. They are considered the oldest present metazoan (600 Ma) [13]. Four classes of extant sponges are currently accepted: Demospongiae, Hexactinellida, Homoscleromorpha, and Calcarea.

The class Calcarea Bowerbank, 1864, consists of a monophyletic group of marine sponges whose skeleton is formed by Mg-calcite spicules, while the others have spicules made of silica [14]. This class is composed of two subclasses, both monophyletic—Calcinea Bidder, 1898, and Calcaronea Bidder, 1898—which differ by cytological, embryological, ontogenetic and skeleton characteristics. In the Calcinea subclass, spicules are mainly regular (equiangular and equiradiate) and the first spicule to be synthesized during ontogeny is the triactine, while in Calcaronea, spicules are irregular and diactines are the first spicules to be produced. Moreover, the larvae in Calcinea is the calciblastula and the nucleus of the choanocytes is basal, while in Calcaronea the larvae is the amphiblastula and the nucleus of the choanocytes is apical. Calcareous sponges are considered viviparous and their larvae are lecytotrophic. Calcinea has two orders, Clathrinida Hartman, 1958, and Murrayonida Vacelet, 1981, the former being the most diverse. The genera *Leucetta* Haeckel, 1872, and *Clathrina* Gray, 1867, are part of the Clathrinida order (see Supplementary Data and Supplementary Figures S1–S4, and Supplementary Table S1 for detailed description and phylogeny of *Leucetta* and *Clathrina* species).

### 2.2. Polyandrocarpamines Are Potent Inhibitors of the DYRK and CLK Kinases

Leucettamine B [8] shares a 2-aminoimidazolone scaffold with various marine natural products such as polyandrocarpamines [15,16], dispacamide [17,18,19], aplysinopsine [20,21], clathridine/clathridimine [8,22], hymenialdisine/spongiacidin B [23,24,25,26,27,28,29,30] and phorbatopsin [31,32] (Figure 1). A summary of all marine aminoimidazolones described so far, and their natural sources and structures, is presented in Supplementary Table S2 and Supplementary Figure S5.

We tested a selection of these 2-aminoimidazolones (Figure 1) on a battery of 27 purified kinases (14 mammalian and 13 unicellular parasites (Supplementary Table S3)). Dose-response curves run with the active compounds provided IC_50_ values that are reported in Table 1. As expected [29,30], hymenialdisine and the closely related spongiacidin B were very potent inhibitors of most kinases (IC_50_ values in the 1–10 nM range). Yet they were not very selective, inhibiting essentially all tested kinases. With the exception of PfGSK-3, the *Plasmodium falciparum* orthologue of glycogen synthase kinase 3 (GSK-3) [33], and LmCK1, the *Leishmania major* orthologue of casein kinase 1 (CK1) [34], unicellular parasite kinases tended to be less sensitive than their mammalian counterparts.

**Figure 2:**
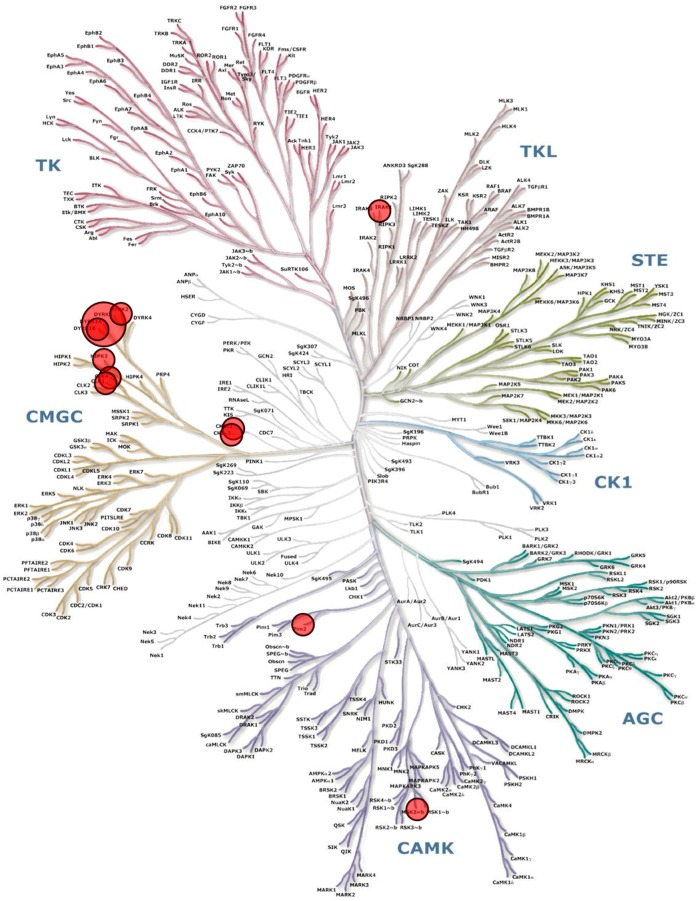
TREEspot™ kinase interaction map of polyandrocarpamine A with 442 human kinases (DiscoveRx KinomeScan^®^, San Diego, CA, USA). Polyandrocarpamine A was tested at a 1 μM final concentration in the kinase interaction panel. A semi-quantitative scoring of this primary screen was estimated. This score relates to a probability of a hit rather than strict affinity. Scores >10, between 1–10 and <1 indicate that the probability of being a false positive is <20%, <10%, <5%, respectively. Results are presented in Table 2 and here as a TREEspot™ kinase interaction maps. Circles indicate the major hits and their size is proportional to the scores. All 442 values are provided in the Supplementary Table S4.

Aplysinopsine, dispacamide, clathridine and clathridimine were essentially inactive in the kinase panel. As expected [6], leucettamine B was modestly active (sub-micromolar IC_50_ values), and leucettine L41 was potent on members of the DYRKs and CLKs families. Again, little inhibitory activity was detected with kinases from unicellular parasites. Finally, both polyandrocarpamines A and B were found to display significantly selective inhibitory effects on DYRKs and somewhat less on CLKs (Table 1). To investigate the selectivity of polyandrocarpamines, polyandrocarpamine A was screened on the large-scale DiscoveRx KinomeScan panel (Figure 2, Supplementary Table S4). This interaction assay provides a semi-quantitative scoring view of the affinity of a compound for any of 442 human kinases [35]. This comprehensive screening approach confirmed the rather good selectivity of polyandrocarpamine A for DYRKs, and detected additional affinity for CSNK2A1/A2, CLK1/4 (Table 2).

**Figure 3:**
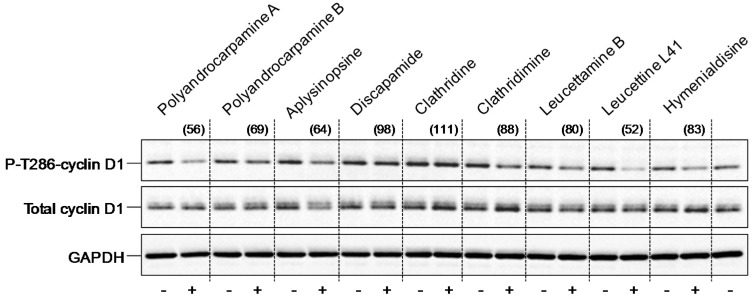
Inhibition of cyclin D1 Thr286 phosphorylation. SH-SY5Y cells expressing human DYRK1A were treated for 24 h with dimethylsulfoxide (DMSO) (−) or 30 μM of each compound (+) (3 μM for leucettine L41 and hymenialdisine). Proteins were resolved by SDS-PAGE and analyzed by Western blotting with antibodies directed against P-T286-cyclin D1, total cyclin D1 or glyceraldehyde-3-phosphate dehydrogenase (GAPDH) (used as loading control). Numbers in parentheses indicate the level of cyclin D1 phosphorylation relative to that of control, DMSO-treated cells (100%).

To confirm the effects of the active products on native DYRK1A in a cellular context, we made use of a SH-SY5Y neuroblastoma cell line expressing human DYRK1A [36] and antibodies directed against cyclin D1 phosphorylated at Threonine 286, a reported DYRK1A phosphorylation site [36,37] (Figure 3). SH-SY5Y-DYRK1A cells were exposed for 24 h to each product (30 μM, except for hymenialdisine and leucettine L41, which were tested at 3 μM), cells were harvested and their proteins resolved by SDS-PAGE, followed by Western blotting with antibodies against P-Thr286-cyclin D1, total cyclin D1 and GAPDH (loading control) (Figure 3). Phosphorylation of cyclin D1 at Thr286 was partially inhibited by polyandrocarpamine A (better than polyandrocarpamine B, leucettine L41 and hymenialdisine, confirming the potent effects on DYRK1A in a cellular context.

**Figure 4:**
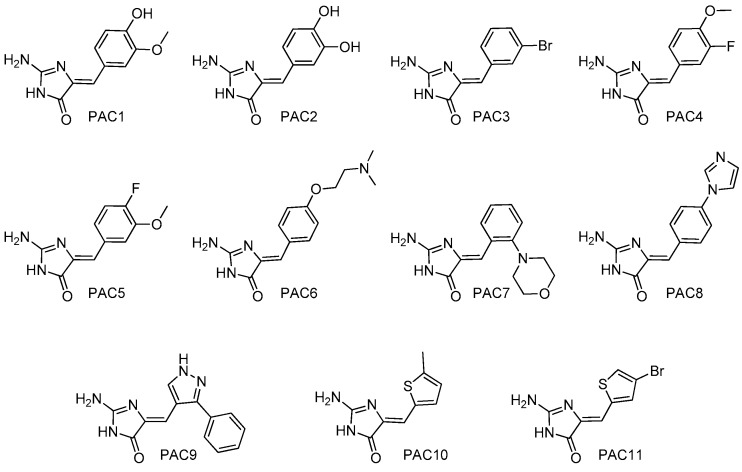
Structure of synthetic analogues and derivatives of polyandrocarpamines (PAC1-PAC11) used in this study.

To extend these results we synthesized both polyandrocarpamines, as well as a series of derivatives [38] (Figure 4), and tested them on a panel of 16 purified kinases. Synthesized (Table 3, PAC 1 and 2) and natural (Table 3, PAC 12 and 13) polyandrocarpamines showed very similar effects on the target kinases. In contrast, none of the synthetic analogues displayed significant efficacy in this kinase panel.

## 3. Discussion

Calcareous sponges of the *Leucetta and Clathrina* genera and a few other marine invertebrates produce a wealth of related yet diverse 2-aminoimidazolone alkaloids (Supplementary Table S2 and Supplementary Figure S5). The existence of these alkaloids in several Calcareous sponges raises several questions regarding their production. What is/are the biosynthetic pathway/s involved (see reference 19 for first hypothesis)? Are these molecules actually produced by the sponges or by associated microorganisms? In addition, these molecules may derive from a plankton source taken up (and possibly metabolized) by these filter-feeders. The fact that polyandrocarpamines were identified in ascidians (*Polyandrocarpa* sp.) [15] and that most 2-aminoimidazolones were discovered in calcareous sponges of the *Leucetta* and *Clathrina* genera (Supplementary Table S2) supports the hypothesis of microorganism-derived metabolites. However, cellular localization of clathridimine revealed its presence in sponge cells rather than in associated bacteria [22]. Several 2-aminoimidazolones were found in *Notodoris* nudibranchs and in the calcareous sponges they crawl and feed on [39,40,41]. Altogether these scattered results suggest possible transfers of 2-aminoimidazolones, their precursors and/or metabolites from one organism to another.

Many questions remain open regarding the physiological/ecological functions of these natural products. Are they used as repellant for predators or to control the expansion of associated microorganisms? Do they have specific functions for the sponge/ascidian/nudibranch metabolism itself? What are their natural molecular and cellular targets? Are they synthesized as kinase inhibitors or do they target other enzymes or structural proteins? Some of these may have orthologues in humans. The biological source, functions and natural targets of marine 2-aminoimidazolone alkaloids should be further investigated.

Only a few of these 2-aminoimidazolones have been chemically synthesized (Supplementary Table S2, right column) and are thus relatively easy to access for biological studies of their pharmacological properties. Nevertheless, they have already proved to be promising scaffolds for the design of potential therapeutic drug candidates. The identification of leucettamine B [6] and polyandrocarpamines A and B [this article] as potent and relatively selective inhibitors of disease-relevant kinases opens the way to the development of drug candidates for various applications [7,42]. This could be guided by the crystal structures of leucettines in complex with various kinases which have been solved [7]. At ManRos Therapeutics we are currently developing leucettines as DYRK1A inhibitory drug candidates [6,7,42,43] to address cognitive deficits associated with DS [Nguyen et al., in preparation] and AD [44,45], but applications in cancer, inflammation and diabetes treatment should be investigated, too.

Besides kinase inhibition, polyandrocarpamine derivatives have been recently identified as inhibitors of cystathionine β-synthase, another potential therapeutic target for DS [46]. 2-Aminoimidazolones have been tested for evaluation of their antibacterial activity, anti-biofilm, antifungal, anticancer [32,47], anti-tuberculosis, anti-protozoan, antiviral, anti-inflammatory, adrenergic, anti-histaminic, anti-cholinergic, anti-serotoninergic, cardiac, immunosuppressive, leukotriene B_4_ receptor antagonistic [8,48] and AD drug activities [12]. It would be of great interest to run more marine derived 2-aminoimidazolone alkaloids on wider enzyme/cell screening panels to identify novel targets and potentially novel therapeutic applications. Hopefully these molecules will add to the growing list of marine drugs and drug candidates developed in recent years [49,50].

## 4. Experimental Section

### 4.1. DNA Sequencing, Alignment and Phylogenetic Analyses

The methods used for molecular phylogenetic analyses of *Leucetta* and *Clathrina* species are detailed in the Supplementary Data.

### 4.2. Chemistry: Purification or Synthesis of Polyandrocarpamines A and B, and Analogues

For the purification of clathridine and clathridimine, specimens of *Clathrina clathrus* were collected by scuba diving in the northwestern Mediterranean Sea (Marseille, France) between 10 and 15 m depth. Lyophilized samples (4.2 g) were extracted with 1:1 CH_2_Cl_2_/MeOH (3 × 20 mL, sonication for 15 min) at room temperature. The 1:1 CH_2_Cl_2_/MeOH extract was concentrated under reduced pressure to yield a dark brown viscous oil (208.1 mg), which was chromatographed on a C_18_ SPE column in a vacuum chamber (H_2_O, H_2_O/MeOH 1:3, MeOH, CH_2_Cl_2_, 100 mL of each). The fraction eluted with H_2_O/MeOH, 1:3 (47.9 mg) was subjected to semi-preparative reversed-phase HPLC (Gemini C_6_-phenyl 10 × 250 mm) with increasing amounts of CH_3_CN/0.1% formic acid in H_2_O/0.1% formic acid as eluent (flow rate: 5 mL/min, wavelength: 254 and 280 nm) and afforded clathridimine (4.6 mg, 0.11% of sponge dry weight) and clathridine (2.8 mg, 0.07% of sponge dry weight).

PAC1 to 11 were supplied by Rohan A. Davis The synthesis, purification and spectroscopic characterization of this focused polyandrocarpamine library has been described elsewhere [38].

Aplysinopsine and leucettamine B were synthesized according to a previously described protocol [51,52] respectively. Dispacamide was synthesized as described in [53].

**Figure 5:**
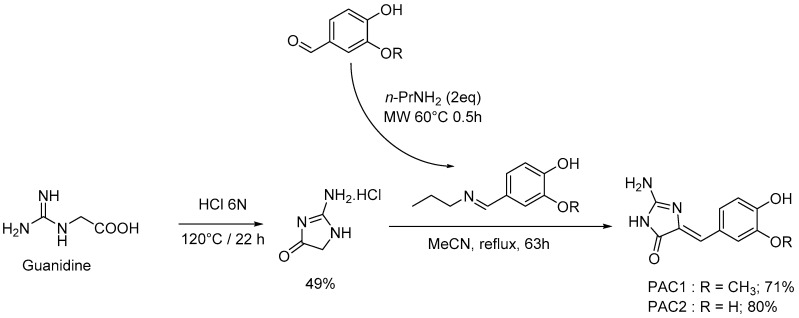
Scheme of the synthesis of polyandrocarpamines.

The synthesis of polyandrocarpamines A & B (PAC1 and PAC2) was performed from corresponding benzaldehyde derivatives, *n*-propylamine and 2-aminoimidazolin-4-one, the preparation of which was previously described in literature [53] (Figure 5). Guanidine (2.95 mmol, 1 eq) and HClaq 6N (9 mL) were heated at 120 °C for 22 h. Water was removed under vacuum and the crude product was solubilized in hot ethanol (9 mL). Diethylether (6 mL) was added, then the mixture was cooled to 0 °C. After 6 h, filtration was performed and the 2-aminoimidazolin-4-one was isolated as a white powder with a 49% yield. RMN ^1^H (D_2_O): δ 4.18 (s, 2H, H-1); RMN ^13^C (D_2_O): δ 48.5 (C-1); 174.9 (C=O); [M − HCl]^+^ = 99.0427 (99.0433 C_3_H_5_N_3_O); F = 200–202 °C. PAC1 and PAC2 were then produced by mixing the corresponding benzaldehyde (vanillin or catechaldehyde 1.48 mmol, 1 eq) with *n*-propylamine (2.95 mmol, 2 eq). The imine formation was performed under microwave irradiation at 60 °C for 30 min (maximized authorized power 60 W). Acetonitrile was added to the crude imine with 2-aminoimidazolin-4-one (1.48 mmol, 1 eq), and the mixture was stirred at reflux for 63 h. The solvent was removed under reduced pressure.

PAC1 was isolated as a beige powder with a 71% yield. RMN ^1^H (DMSO-*d*_6_): δ 3.79 (s, 3H, OCH_3_); 6.23 (s, 1H, C=CH); 6.76 (d, 1H, *J* = 8.0 Hz, H-5); 7.10 (sl, 2H, NH_2_); 7.35 (sl, 1H, H-6); 7.56 (sl, 1H, H-2); 9.24 (sl, 1H, OH); 10.38 (sl, 1H, NH); RMN ^13^C (DMSO-*d*_6_): δ 56.2 (OCH3); 114.6 (C-2); 116.0 (C-5); 124.0 (C=CH); 127.6 (C-6); 147.1 (C-3); 147.9 (C-4); [M]^+^ = 233.0797 (233.0800 C_11_H_11_N_3_O3); F > 260 °C.

PAC2 was isolated as a beige powder with a 80% yield. RMN ^1^H (DMSO-*d*_6_): δ 6.14 (s, 1H, C=CH); 6.70 (d, 1H, *J* = 8.1 Hz, H-5); 6.92 (s, 2H, NH_2_); 7.27 (d, 1H, *J* = 7.5 Hz, H-6); 7.53 (s, 1H, H-2); 8.81 (s, 1H, NH); 9.19 (s, 1H, OH); 10.45 (s, 1H, OH); RMN ^13^C (DMSO-*d*_6_): δ 112.5 (C=CH); 116.6 (C-5); 118.2 (C-2); 123.1 (C-6); 128.2 (C-1); 146.0 (C-3); 146.6 (C-4); F > 260 °C.

### 4.3. Biology

#### 4.3.1. Buffers

Homogenization buffer: 25 mM MOPS; 15 mM EGTA; 15 mM MgCl_2_; 60 mM *β*-glycerophosphate; 15 mM *p*-nitrophenylphosphate; 2 mM dithiothreitol (DTT); 1 mM Na3VO4; 1 mM NaF; 1 mM di-sodium phenylphosphate; 1X protease inhibitor cocktail; 0.2% Nonidet P-40 substitute.

Buffer A: 10 mM MgCl_2_; 1 mM EGTA (MW 380.4); 1 mM DTT (MW 154.2); 25 mM Tris/HCl (MW 121.1), and; 50 μg/mL heparin.

Buffer C: 60 mM *β*-glycerophosphate; 30 mM *p*-nitrophenylphosphate; 25 mM MOPS pH 7.0; 5 mM EGTA; 15 mM MgCl_2_; 1 mM DTT, and; 0.1 mM sodium vanadate.

All chemicals were purchased from Sigma Aldrich (St. Quentin Fallavier, France), unless otherwise stated and the protease inhibitor cocktail was from Roche (Boulogne-Billancourt, France).

#### 4.3.2. Production and Purification of the Parasitic Kinases

The parasitic kinase genes (Supplementary Data, Table S3) were optimized for expression in *E. coli*, synthesized, and cloned in pGEX-6P-1 (GE Healthcare, Sigma-Aldrich, St. Quentin Fallavier, France) with the type II restriction enzymes *BamHI* and *XhoI* for glutathione S-transferase (GST) fusion at the N-terminus (GenScript). Plasmids were transformed into chemically competent *E. coli* BL21(DE3), which were grown overnight at 37 °C in 2 × YT medium containing 100 μg/mL ampicillin. These cultures were used to inoculate 1 L volumes of 2 × YT medium (containing 100 μg/mL ampicillin) in 5 L flasks. The cultures were allowed to grow at 37 °C before the temperature was decreased to 18 °C. At an optical density at 600 nm (OD600) of about 1.0, protein expression was induced overnight at 18 °C with 0.1 mM isopropyl β-d-1-thiogalactopyranoside (IPTG). The bacteria were harvested by centrifugation and were frozen at −20 °C.

Cells were resuspended in a lysis buffer (PBS pH 7.4, 1% NP40, 1 mM DTT, 1 mM EDTA, 1 mM PMSF) in the presence of protease inhibitor cocktail (Roche), and lysozyme was added to 1 mg/mL and incubated 1 h at 4 °C with gentle agitation. Cells were then sonicated on ice before 6 mM MgCl_2_ and 25 U/mL benzonase (Novagen, Merck Millipore, Fontenay sous Bois, France) were added to degrade nucleic acids and decrease sample viscosity. After centrifugation at 4 °C, the soluble fraction was collected and proteins were bound to Glutathione sepharose 4B beads (GE Healthcare). Beads were washed three times with lysis buffer and once with modified buffer C (25 mM MOPS pH 7.0, 60 mM β-glycerophosphate, 15 mM *p*-nitrophenylphosphate, 15 mM EGTA, 15 mM MgCl_2_, 2 mM DTT, 1 mM sodium vanadate). Proteins were eluted with elution buffer (buffer C containing 30 mM reduced glutathione, 15% glycerol, and pH adjusted to 8.5).

#### 4.3.3. Protein Kinase Assays

Kinase activities were assayed in buffer A or C at 30 °C at a final ATP concentration of 15 μmol/L. Blank values were subtracted and activities were expressed in percent of the maximal activity, i.e., in the absence of inhibitors. Controls were performed with appropriate dilutions of DMSO.

The GS-1, CKS, CDK7/9 tide and RS peptide substrates were obtained from Proteogenix (Oberhausbergen, France).

CDK1/cyclin B (M phase starfish oocytes, native), CDK2/cyclin A and CDK5/p25 (human, recombinant) were prepared as previously described [6,7]. Their kinase activity was assayed in buffer A, with 1 mg histone H1/mL, in the presence of 15 μmol/L [γ-^33^P] ATP (3000 Ci/mmol; 10 mCi/mL) in a final volume of 30 μL. After 30 min incubation at 30 °C, the reaction was stopped by harvesting onto P81 phosphocellulose supernatant (Whatman, Dutscher SAS, Brumath, France) using a FilterMate harvester (PerkinElmer, Courtaboeuf, France) and were washed in 1% phosphoric acid. Scintillation fluid was added and the radioactivity measured in a Packard counter.

CDK9/cyclin T (human, recombinant, expressed in insect cells) was assayed as described for CDK1/cyclin B, but using CDK7/9 tide (YSPTSPSYSPTSPSYSPTSPSKKKK) (8.1 μg/assay) as a substrate.

GSK-3α/β (porcine brain, native) and PfGSK3 (*plasmodium falciparum*, recombinant, expressed in *E. coli* as GST fusion proteins) was assayed, as described for CDK1 with 0.5 mg BSA/mL + 1 mM DTT, using GS-1 (YRRAAVPPSPSLSRHSSPHQSpEDEEE) (pS stands for phosphorylated serine), a GSK-3 specific substrate [54].

CK1δ/ε (porcine brain, native) and *LmCK1* (leishmania major, recombinant, expressed in *E. coli* as HIS fusion proteins [34]) was assayed as described for CDK1 but in buffer C and using 25 μM CKS peptide (RRKHAAIGpSAYSITA), a CK1-specific substrate [55].

DYRK1A, 1B, 2 and 3 (Human, recombinant, expressed in *E. coli* as GST fusion proteins) were purified by affinity chromatography on glutathione-agarose and assayed as described for CDK1/cyclin B with with 0.5 mg BSA/mL + 1 mM DTT and using Woodtide (KKISGRLSPIMTEQ) (1.5 μg/assay) as a substrate, a residue of transcription factor FKHR.

CLK1, 2, 3 and 4 (mouse, recombinant, expressed in *E. coli* as GST fusion proteins) were assayed as described for CDK1/cyclin B with 0.5 mg BSA/mL + 1 mM DTT and RS peptide (GRSRSRSRSRSR).

Protozoan DYRKs and CLKs (recombinant, expressed in *E. coli)* were assayed as described for CDK1/cyclin B with 0.5 mg BSA/mL + 1 mM DTT and Woodtide or RS peptide for DYRKs or CLKs isoforms.

#### 4.3.4. Kinase Interaction Panel (Ambit Biosciences/DiscoveRx)

Assays were performed essentially as described previously [35]. For most assays, kinase-tagged T7 phage strains were grown in parallel in 24-well blocks in an *E. coli* host derived from the BL21 strain. *E. coli* were grown to log-phase and infected with T7 phage from a frozen stock (multiplicity of infection ~0.1) and incubated with shaking at 32 °C until lysis (~90 min). The lysates were centrifuged (6000× *g*) and filtered (0.2 μm) to remove cell debris. The remaining kinases were produced in HEK-293 cells and subsequently tagged with DNA for qPCR detection. Streptavidin-coated magnetic beads were treated with biotinylated small molecule ligands for 30 min at room temperature (RT) to generate affinity resins for kinase assays. The liganded beads were blocked with excess biotin and washed with blocking buffer (SeaBlock (Pierce, ThermoFisher Scientific, Illkirch, France), 1% BSA, 0.05% Tween 20, 1 mM DTT) to remove unbound ligand and to reduce non-specific phage binding. Binding reactions were assembled by combining kinases, liganded affinity beads, and test compounds in 1× binding buffer (20% SeaBlock, 0.17× PBS, 0.05% Tween 20, 6 mM DTT). An 11-point threefold serial dilution of each test compound was prepared in 100% DMSO at 100× final test concentration which and subsequently diluted to 1× in the assay. All reactions were performed in polystyrene 96-well plates in a final volume of 0.135 mL. The assay plates were incubated at RT with shaking for 1 h and the affinity beads were washed four times with wash buffer (1× PBS, 0.05% Tween 20). The beads were then resuspended in elution buffer (1× PBS, 0.05% Tween 20, 0.5 μM non-biotinylated affinity ligand) and incubated at RT with shaking for 30 min. The kinase concentration in the eluates was measured by qPCR.

#### 4.3.5. Thr286-Cyclin D1 Phosphorylation in SH-SY5Y Cells

Cell treatments. SH-SY5Y cells conditionally expressing human DYRK1A [36], under the control of doxycycline (gift from Dr. Walter Becker) were cultured Dulbecco’s Modified Eagle Medium (DMEM): Nutrient Mixture F-12 (DMEM/F-12, Gibco, c/o Invitrogen, Saint Aubin, France) containing 1% Penicillin Streptomycin mixture (Gibco) and 10% fetal bovine serum (FBS, Gibco) in a humidified, 5% CO_2_ incubator at 37 °C. Cells were split routinely once a week. They were first rinsed with phosphate buffered saline (PBS, Gibco) and detached from the plate bottom using 2 mL Trypsin (Gibco) at 37 °C for 3–4 min. Eight mL of fresh medium were added to the cell suspension and the mix was centrifuged for 3 min at 1000 rpm. The cell pellet was resuspended in fresh medium before seeding (½ dilution) in new 150 mm plates. First, 1 μg/mL doxycycline was used to induce DYRK1A expression and cells were incubated 24 h in a humidified, 5% CO_2_ incubator at 37 °C. Drugs (0, 3 or 30 μM final) were then added with a final concentration of 0.1% DMSO and cells were incubated for an additional 24 h in a humidified, 5% CO_2_ incubator at 37 °C. Afterwards, cells were scrapped in medium and centrifuged for 5 min at 3000 rpm at 4 °C. The pellets were washed twice with PBS, centrifuge for 5 min at 10,000 g at 4 °C, snap-frozen in liquid nitrogen and kept at −80 °C.

Western blot analysis. Cell pellets were lysed in homogenization buffer. Protein extracts were mixed (1:1 *v*/*v*) with sample buffer (2× NuPAGE LDS sample buffer, 200 mM DTT) and 20 μg of total proteins were loaded on NuPAGE 4–12% Bis-Tris protein gels. Electrophoresis was run at 70 V in MOPS buffer for 4 h. Rapid blot transfers were performed at 2.5 A/25 V for 7 min. Blotting membranes were blocked in milk (5% Regilait in Tris Buffered Saline with 0.1% Tween (TBST)) for 1 h. Blotting membranes were incubated overnight at 4 °C with anti-cyclin D1 antibody (1:2000 dilution; Cell Signaling, Leiden, The Netherlands) or anti-phospho T286-cyclin D1 antibody (1:2000 dilution; Cell Signaling) or for 1 h at RT with anti-DYRK1A antibody (1:2000 dilution; Abnova, Taoyuan City, Taiwan) or anti-GAPDH antibody (1:30,000 dilution; Bio-Rad, Marnes-la-Coquette, France). Next, blotting membranes were incubated for 1 h at RT with goat anti-rabbit or goat anti-mouse antibodies (Bio-Rad). Chemiluminescent detection was achieved with homemade ECL-Tris buffer (100 mM Tris pH 8.5, 0.009% H_2_O_2_, 0.225 mM *p*-coumaric acid, 1.25 mM luminol) with Fusion F × 7 camera software.

## Figures and Tables

**Table 1:** Protein kinase selectivity of a selection of 10 marine natural products sharing the 2-aminoimidazolone scaffold: leucettamine B and its synthetic leucettine L41 derivative; polyandrocarpamines A & B, aplysinopsine, dispacamide, hymenialdisine, spongiacidin B, clathridine, and clathridimine. All compounds were tested at various concentrations against 27 purified kinases (14 mammalian and 13 unicellular parasites). IC_50_ values (concentrations inducing 50% inhibition of maximal, non-inhibited kinase activity), calculated from the dose-response curves, are reported in μM. IC_50_ values below 1 μM are shown in bold.

Kinases	Leucettamine B	Leucettine L41	Polyandrocarpamine A	Polyandrocarpamine B	Aplysinopsine	Dispacamide	Hymenialdisine	Spongiacidin B	Clathridine	Clathridimine
CDK1/cyclin B	>10	>10	>10	>10	>10	>10	**0.005**	**0.031**	>10	>10
CDK2/cyclin A	>10	>10	>10	>10	>10	>10	**0.011**	**0.068**	>10	>10
CDK5/p25	>10	>10	>10	>10	>10	>10	**0.0061**	**0.042**	>10	>10
CDK9/cyclin T	>10	>10	**0.93**	5.2	>10	>10	**0.011**	**0.2**	>10	>10
CK1δ/ε	>10	4.2	3.3	>10	>10	>10	**0.0037**	**0.031**	>10	>10
CLK1	**0.1**	**0.039**	1.0	2.7	>10	>10	**0.003**	**0.022**	6.9	7.2
CLK2	**0.91**	**0.21**	1.2	4.9	>10	>10	**0.0041**	**0.0073**	6.0	6.1
CLK3	9.7	2.4	2.6	8.6	>10	>10	**0.0053**	**0.049**	>10	>10
CLK4	**0.12**	**0.031**	**0.32**	**0.82**	>10	>10	**0.0029**	**0.0036**	3.9	3.5
DYRK1A	**0.42**	**0.032**	**0.27**	**0.47**	>10	>10	**0.0033**	**0.78**	7.8	5.2
DYRK1B	1.8	**0.05**	**0.43**	1.2	>10	>10	**0.0032**	**0.0058**	>10	5.6
DYRK2	**0.49**	**0.088**	**0.42**	**0.76**	>10	>10	**0.0031**	**0.021**	7.1	6.2
DYRK3	**0.6**	**0.42**	**0.17**	**0.88**	>10	>10	**0.0049**	**0.044**	>10	6.1
GSK-3α/β	7.3	1.1	>10	>10	>10	>10	**0.0032**	**0.044**	>10	>10
PfGSK-3	>10	8.5	>10	>10	>10	>10	**0.0041**	**0.038**	>10	>10
PfCLK1	**0.79**	**0.42**	**0.65**	3.1	>10	>10	**0.011**	**0.085**	>10	>10
LmCK1	>10	>10	>10	>10	>10	>10	**0.0025**	**0.025**	>10	>10
LmCLK	>10	>10	>10	>10	>10	>10	**0.4**	5.2	>10	>10
LmDYRK2	4.2	2.9	5.9	>10	>10	>10	**0.38**	9.0	>10	>10
LdDYRK1B	6.9	**0.82**	1.1	>10	>10	>10	**0.042**	**0.5**	>10	>10
LdDYRK3	>10	>10	>10	>10	>10	>10	**0.021**	**0.3**	>10	7.9
LdDYRK4	>10	>10	>10	>10	>10	>10	>10	>10	>10	>10
TbCLK1	>10	>10	>10	>10	>10	>10	**0.53**	**0.71**	>10	>10
TcCLK1	>10	>10	>10	>10	>10	>10	0.51	1.1	>10	>10
CpLAMMER	1.8	0.1	0.17	0.2	>10	>10	0.016	0.13	>10	>10
GlCLK	3.1	>10	0.95	2.2	>10	>10	0.012	0.12	>10	>10
TgCLK	4.1	>10	>10	>10	>10	>10	0.041	0.48	>10	>10

**Table 2:** Protein kinase selectivity of polyandrocarpamine A in a kinase interaction assay (DiscoveRx KinomeScan^®^). Polyandrocarpamine A was tested at 1 μM on a 442 kinases interaction panel. A semi-quantitative scoring of this primary screen was obtained. This score relates to a probability of a hit rather than strict affinity. Scores >10, between 1–10 and <1 indicate that the probability of being a false positive is <20%, <10%, <5%, respectively. The 11 best scores are presented. Full results are available in Supplementary Table S4.

Kinases	Abbreviation	Score
Dual specificity tyrosine-phosphorylation-regulated kinase 1A	DYRK1A	3
Casein kinase 2 α	CSNK2A1	12
Homeodomain interacting protein kinase 3	HIPK3	19
Proviral Integrations of Moloney virus 2	PIM2	21
Ribosomal protein S6 kinase alpha-4 (Kin.Dom.2-C-terminal)	RPS6KA4	22
Dual specificity tyrosine-phosphorylation-regulated kinase 2	DYRK2	24
Dual specificity tyrosine-phosphorylation-regulated kinase 1B	DYRK1B	25
Casein kinase 2 α’	CSNK2A2	26
Interleukin-1 receptor-associated kinase 1	IRAK1	28
Cdc2-like kinase 4	CLK4	30
Cdc2-like kinase 1	CLK1	32

**Table 3:** Protein kinase selectivity of synthetic products derived from polyandrocarpamines A and B. All compounds were tested at various concentrations on 16 purified kinases (14 mammalian and two unicellular parasites). IC_50_ values, calculated from the dose-response curves, are reported in μM. IC_50_ values below 1 μM are shown in bold. PAC1 and PAC2, PAC12 and PAC13 are synthetic and natural polyandrocarpamines A and B, respectively.

Kinases	Polyandrocarpamines (PAC)												
	1	2	3	4	5	6	7	8	9	10	11	12	13
CDK1/cyclin B	>10	>10	>10	>10	>10	>10	>10	>10	>10	>10	>10	>10	>10
CDK2/cyclin A	>10	>10	≥10	≥10	>10	>10	>10	>10	>10	>10	>10	>10	>10
CDK5/p25	>10	>10	>10	>10	>10	>10	>10	>10	>10	>10	>10	>10	>10
CDK9/cyclin T	1.1	2	>10	>10	≥10	>10	>10	**0.51**	>10	>10	>10	**0.9**	1.8
CK1δ/ε	5.2	>10	≥10	≥10	>10	>10	>10	>10	3.6	>10	>10	5	>10
CLK1	**0.43**	1	2.2	≥10	2.2	>10	>10	>10	≥10	>10	4.2	**0.47**	**0.59**
CLK2	1.2	2.5	6.3	>10	7.5	>10	>10	>10	>10	>10	≥10	**0.64**	2.42
CLK3	2.3	9.2	>10	>10	>10	>10	>10	>10	>10	>10	>10	2.7	10
CLK4	**0.4**	**0.95**	2.3	≥10	2.4	>10	>10	>10	>10	>10	3.2	**0.47**	**0.91**
DYRK1A	**0.23**	**0.75**	2.1	4	1.3	>10	>10	>10	>10	3	2.1	**0.3**	**0.59**
DYRK1B	**0.4**	1.1	8.2	>10	6.1	>10	>10	>10	>10	>10	6.2	**0.47**	**0.96**
DYRK2	**0.14**	**0.48**	2.2	4.5	**0.64**	>10	>10	>10	>10	3.4	1.3	**0.24**	**0.38**
DYRK3	**0.28**	**0.9**	>10	5.1	5.7	>10	>10	>10	>10	>10	2.4	**0.34**	**0.68**
GSK-3α/β	>10	>10	>10	>10	>10	>10	>10	>10	>10	>10	2	>10	>10
PfGSK-3	>10	>10	>10	>10	>10	>10	>10	>10	>10	>10	>10	>10	>10
LmCK1	>10	>10	>10	>10	>10	>10	>10	>10	**0.63**	>10	>10	>10	>10

## Supplementary Materials

This material is available free of charge via the internet at www.mdpi.com/1660-3397/15/10/316/s1.

## Abbreviations

**AD**, Alzheimer’s disease; **CDKs**, cyclin-dependent kinases; **CK1**, casein kinase 1; **CK2**, casein kinase 2; **CLKs**, cdc2-like kinases; **DMEM**, Dulbecco’s Modified Eagle Medium; **DMSO**, dimethylsulfoxide; **DS**, Down syndrome; **DTT**, dithiothreitol; **DYRKs**, dual-specificity, tyrosine phosphorylation regulated kinases; **GAPDH**, glyceraldehydes-3-phosphate dehydrogenase; **GSH**, glutathione; **GSK-3**, glycogen synthase kinase-3; **GST**, glutathione-S-transferase; **IC50**, concentration inducing 50% inhibition of the maximal kinase activity; **IPTG**, isopropyl β-D-1-thiogalactopyranoside; **PBS**, phosphate-buffered saline; **RT**, room temperature.
