# Supplementary material for: Marine-Derived 2-Aminoimidazolone Alkaloids. Leucettamine B-Related Polyandrocarpamines Inhibit Mammalian and Protozoan DYRK & CLK Kinases
Source: Mar Drugs. 2017 Oct 17;15(10):316. doi: 10.3390/md15100316 (PMC5666424; doi:10.3390/md15100316)
Supplement: Supplementary file 1 [file marinedrugs-15-00316-s001.pdf]

Marine Drugs
ISSN 1660-3397
© 2008 by MDPI
www.mdpi.org/marinedrugs

SUPPLEMENTARY DATA

Marine Derived 2-Aminoimidazolone Alkaloids. Leucettamine B-Related Polyandrocarpamines Inhibit Mammalian and Protozoan DYRK & CLK Kinases.

Nadège **LOAËC**, Eletta **ATTANASIO**, Benoît **VILLIERS**, Emilie **DURIEU**, Tania **TAHTOUH**, Morgane **CAM**, Rohan A. **DAVIS**, Aline **ALENCAR**, Mélanie **ROUE**, Marie-Lise **BOURGUET-KONDRACKI**, Peter **PROKSCH**, Emmanuelle **LIMANTON**, Solène **GUIHENEUF**, François **CARREAUX**, Jean-Pierre **BAZUREAU**, Michelle **KLAUTAU** and Laurent **MEIJER**

SUPPLEMENTARY RESULTS
Phylogeny of calcareous sponges, with special emphasis of *Leucetta* and *Clathrina* 2

SUPPLEMENTARY MATERIAL & METHODS 3

SUPPLEMENTARY TABLES
Supplementary Table S1. Analysed specimens 4
Supplementary Table S2. Aminoimidazole alkaloids from marine invertebrates 5
Supplementary Table S3. Kinase orthologues from unicellular parasites 6
Supplementary Table S4. DiscoveRx KinomeScan® kinase selectivity panel 7

SUPPLEMENTARY FIGURES
Supplementary Figure S1. *Leucetta microraphis* 12
Supplementary Figure S2. *Leucetta chagosensis* 13
Supplementary Figure S3. *Clathrina clathrus* 14
Supplementary Figure S4. Maximum likelihood tree 15
Supplementary Figure S5. Structure of aminoimidazole alkaloids
16

SUPPLEMENTARY REFERENCES 20

SUPPLEMENTARY RESULTS

Phylogeny of calcareous sponges, with special emphasis of *Leucetta* and *Clathrina*.

Given the importance of Leucetta and Clathrina in the production of various 2-aminoindazolinones, we provide here a full description of both genera and the three major species that have been investigated for 2-aminoindazolinones, namely *Leucetta microraphis* (Figure S1), *Leucetta chagosensis* (Figure S2) *and Clathrina clathrus* (Figure S3). We also provide a phylogenetic tree of all species that have been described in the Clathrinida order (Figure S4).

1. *Leucetta* Haeckel 1872

The family Leucettidae de Laubenfels, 1936 reunites two genera, *Leucetta* and *Pericharax* Poléjaeff, 1883, which are differentiated only by the presence of subcortical lacunae in the latter. Therefore, both *Pericharax* and *Leucetta* are sponges with a solid body and leuconoid aquiferous system. The choanoskeleton is well-developed and composed of regular triactines and/or tetractines (Borojevic *et al*., 2002). *Leucetta* is a very abundant and cosmopolitan genus whose species are mainly distributed in tropical seas (Borojevic *et al*., 2002). They are massive and more conspicuous than most calcareous sponges.

Leucetta microraphis Haeckel, 1872 (Figure S1)

Leucetta microraphis has already been reported as white, green (Dendy, 1892) and brownish-yellow or greenish-yellow (Wörheide & Hooper, 1999) in life (Figure S1, G). After fixation it becomes white to dark beige (Figure S1, A). Its body is massive and amorphous with single or multiple apical naked oscula. Surface is hispid due to the presence of many colossal triactines and texture is friable. The atrial cavity is reduced and the aquiferous system is leuconoid, with rounded choanocytary chambers. There is no differentiation between the cortical and the choanosomal skeletons. The cortical skeleton is thin and composed of colossal and abundant triactines tangentially disposed over small triactines. The choanoskeleton is dense, composed mainly of small triactines but also of several colossal triactines. Small tetractines surround the large exhalant canals and project their apical actines into them. The atrial skeleton is composed mainly of small triactines, but colossal triactines and small tetractines are also present.

SPICULES

Small triactines (Figure S1, D): regular (equiradiate and equiangular). Actines are conical with sharp tips (120-193.7 (±27.1)-250/ 20-20 (±0)-20 µm), n=30 (syntype PMJ 133).

Colossal triactines (Figure S1, E): regular (equiradiate and equiangular). Actines are conical with sharp tips (655-1,036 (±239.3)-1,450/ 75-158.3 (±47)-225 µm), n=30, (syntype PMJ 133).

Small tetractines (Figure S1, F): regular (equiradiate and equiangular). Actines are conical with sharp tips (140-162.7 (±27.8)-280/ 20-20 (±0)-20 µm), n=30, (syntype PMJ 133). The apical actine is shorter and thinner than the basal ones. It is cylindrical and slightly curved near the tip.

HABITAT AND DISTRIBUTION

Leucetta microraphis is a common species in the Indo-Pacific, where it can be found in exposed and light-protected areas, such as crevices and steep walls (Wörheide & Hooper, 1999). The distribution of *L. microraphis* has already been considered cosmopolitan, including the Indo-Pacific, Atlantic, Mediterranean, Red Sea and Antarctica (see Wörheide & Hooper, 1999). However, in 2009, Valderrama *et al*. (2009) showed that what had been called *L. microraphis* in the Atlantic was in fact *L. floridana*. Presently, *L. microraphis* is considered to be present only in the Indo-Pacific, but even this more restricted distribution may still be hiding other species. Its currently accepted distribution is Australia, New Zealand, New Caledonia, French Polynesia and Madagascar. Although Haeckel (1872) had not made clear where the type locality of *Leucetta primigenia* var. *microraphis* is, since Lendenfeld (1885) elevated it to the species category, Australia is considered as its type locality.

Leucetta chagosensis Dendy, 1913 (Figure S2)

Leucetta chagosensis (the "lemon sponge") is bright yellow alive (Figure S2, G) and brown after fixation (Figure S2, A). It is massive and globose, but according to the original description, it can be encrusting. It has one to some few apical oscula surrounded by membrane. Surface is smooth but texture is friable. Subcortical cavities are present. The atrial cavity is reduced and the aquiferous system is leuconoid with sub-spherical choanocytary chambers. The skeleton is composed mainly of small triactines, which are present everywhere, while colossal triactines are present only on the cortex and very rare small tetractines can be found only in the atrium.

SPICULES

Small triactines (Figure S2, D): regular (equiradiate and equiangular). Actines are conical and sharp (100-172.3 (±27.1)-210/ 10-17 (±4.7)-20 μm), n=30 (Holotype, BMNH 1920.12.9.51).
Colossal triactines (Figure S2, E): regular (equiradiate and equiangular). Actines are conical and sharp (330-482.9 (±102.3)-630/ 40-55.7 (±14.8)-80 μm), n=8 (Holotype, BMNH 1920.12.9.51).
Small tetractines (Figure S2, F): regular (equiradiate and equiangular). Actines are conical and sharp (100-120.6 (±22.1)-190/ 10-15 (±3.5)-20 μm), n=25, Holotype. The apical actine is slightly thinner and much shorter than the basal ones (60-68.2 (±8)-80/ 8-8.2 (±0.4)-10 μm), n=4 (Holotype, BMNH 1920.12.9.51).

HABITAT AND DISTRIBUTION

Leucetta chagosensis is a very abundant sponge both in protected and in illuminated areas. It was already reported to the Indian Ocean (Chagos Archipelago - type locality; Madagascar, Northern Red Sea, Japan (Okinawa), Indonesia, Australia (Queensland and Fremantle), New Caledonia and French Polynesia (Dendy, 1913; Wörheide & Hooper, 1999; 2008; Borojevic & Klautau, 2000). Phylogeographical studies showed, however, that populations of this species are highly structured and this wide distribution may be concealing cryptic species (Wörheide *et al*., 2002; 2008).

2. Clathrina Minchin, 1900

Clathrina is the most diverse genus of the family Clathrinidae Minchin, 1900. It comprises delicate clathroid species whose skeleton is composed only of triactines (Rossi *et al*., 2011; Klautau *et al*., 2013). It is a widespread genus, present in all oceans and latitudes, however, most of its species have a restricted distribution.

Clathrina clathrus (Schmidt, 1864) (Figure S3)

Clathrina clathrus is a yellow sponge (Figure S3, A) that becomes beige after fixation. It is a very common species in the Mediterranean and Adriatic Seas (Klautau & Valentine, 2003; Imesek *et al*., 2014). Its body is formed by loosely anastomosed tubes and the few oscula are organised in water-collecting tubes. The aquiferous system is asconoid and the skeleton is composed only of triactines without organisation.

SPICULES

Triactines (Figure S3, B): Regular (equiradiate and equangular). Actines are cylindrical, undulated with rounded tips (85-92 (±4.3)-100/ 7.3 (±0.5) μm), n=20, (Syntype, measurements from Klautau & Valentine, 2003).

HABITAT AND DISTRIBUTION

Clathrina clathrus is a very delicate species that lives in light protected habitats, such as caves and crevices. It has already been considered as a cosmopolitan species, but currently its accepted distribution is restricted to the whole Mediterranean Sea and to the Northwestern coast of the Atlantic. In the past, all yellow clathrinas were identified as *C. clathrus*, however, a genetic study showed that Mediterranean and Southwestern Atlantic populations of yellow clathrinas were not conspecific (Solé-Cava *et al*., 1991). Since then, molecular and morphological studies have proved the existence of several different species of yellow clathrinas in the world (Wörheide & Hooper, 1999; Borojevic & Klautau, 2000). However, all of them can be grouped in the same monophyletic clade (Rossi *et al*., 2011; Klautau *et al*., 2013).

3. Remarks

Clathrina clathrus, *L. microraphis* and *L. chagosensis* group within the subclass Calcinea, but the phylogenetic tree of Calcinea shows three main groups, one that reunites sponges without tetractines (*Clathrina*) and two of sponges with tetractines (all the other genera) (Rossi *et al*., 2011; Klautau *et al*., 2013) (Figure S5). *Clathrina clathrus* groups in the clade of *Clathrina*, more specifically in a clade of yellow clathrinas only. It is a sister group of *C. aurea* from the Western Atlantic (Brazil). *Leucetta microraphis* and *L. chagosensis* group in the clade of *Leucetta*, but separately from each other. *Leucetta microraphis* is more related to *L. potiguar* and *L. floridana* from the Western Atlantic, while *L. chagosensis* is more related to *L. pyriformis* and *L. antarctica*, both from Antarctica.

SUPPLEMENTARY MATERIAL & METHODS

DNA sequencing, alignment and phylogenetic analyses

The analysed region was the internal transcribed spacer (ITS), including 18S partial, ITS1, 5.8S, ITS2 and 28S partial. All sequences were obtained from the GenBank (Table S1). Sequences were aligned with the Q option of the MAFFT program (Katoh & Standley, 2013), which takes into consideration the secondary structure of the ITS. Scoring matrix was PAM/k1/42, gap penalty 1.53 and offser value 1/40. As an appropriate outgroup is not available, we root the tree with the mid-point rooting method. Final alignment contained 1,096 bp.

A maximum likelihood tree was generated using the MEGA 6.0 platform (Tamura *et al*., 2013). The substitution model was the general time reversal (GTR). Partial deletion was chosen and 1,000 bootstrap pseudo-replicates (Felsenstein, 1985) were performed on the ML tree.

SUPPLEMENTARY TABLES

Supplementary Table S1. Analyzed (molecular phylogeny) specimens with collection sites, voucher numbers and GenBank accession numbers.

Species	Collection site	Voucher number	GenBank (ITS)
Arturia cf. *hirsuta*	Cabo Verde	ZMAPor07061	KC843431
Ascaltis reticulum	Mediterranean Sea	UFRJPor6258	HQ588973
Ascandra contorta	Mediterranean Sea	UFRJPor6327	HQ588970
Ascandra corallicola	Norway	UFRJPor6329	HQ588994
Ascandra falcata	Mediterranean Sea	UFRJPor5856	HQ588962
Borojevia aspina	Brazil	UFRJPor5245	HQ588998
Borojevia brasiliensis	Brazil	UFRJPor5214	HQ588978
Borojevia cerebrum	Mediterranean Sea	UFRJPor6322	HQ588964
Clathrina aurea	Brazil	MNRJ5170	HQ588960
Clathrina aurea	Brazil	MNRJ8990	HQ588958
Clathrina aurea	Brazil	MNRJ8998	HQ588968
Clathrina clathrus	Mediterranean Sea	UFRJPor6315	HQ588974
Clathrina clathrus	Mediterranean Sea	UFRJPor6325	HQ588965
Clathrina clathrus	Mediterranean Sea	UFRJPor6326	HQ588972
Clathrina conifera	Brazil	MNRJ8991	HQ588959
Clathrina conifera	Brazil	MNRJ8997	HQ588957
Clathrina coriacea	Norway	UFRJPor6330	HQ588986
Clathrina cylindractina	Brazil	UFRJPor 5413	HQ588993
Clathrina fjordica	Chile	MNRJ 8143	HQ588984
Clathrina luteoculcitella	Australia	QMG 313684	HQ588989
Ernstia sp. nov. 1	Brazil	UFRJPor6621	KC843433
Ernstia sp. nov. 14	Indonesia	ZMAPor08390	KC843451
Ernstia sp. nov. 2	Brazil	UFRJPor6617	KC843434
Ernstia tetractina	Brazil	UFRJPor5183	HQ589000
Leucascus simplex	French Polynesia, Moorea	BMOO16283	KC843454
Leucetta antarctica	Antarctica	MNRJ13798	KC849700
Leucetta chagosensis	Australia	QMG313774	AM850505
Leucetta chagosensis	Australia	QMG313944	AM850528
Leucetta chagosensis	Australia	QMG313946	AM850529
Leucetta floridana	Caribbean	UFRJPor5357	EU781970
Leucetta floridana	Caribbean	UFRJPor5359	EU781969
Leucetta floridana	Caribbean	UFRJPor5360	EU781968
Leucetta microraphis	Australia, Wistari Reef	QMG313659	AJ633874
Leucetta microraphis	Australia, Wistari Reef	QMG315140	AJ633871
Leucetta potiguar	Brazil	UFPEPor547	EU781986
Leucetta potiguar	Brazil	UFPEPor569	EU781987
Leucetta potiguar	Brazil	UFPEPor588	EU781988
Leucetta pyriformis	Antarctica	MNRJ13843	KC843457

Supplementary Table S2. Aminoimidazole alkaloids from marine invertebrates: natural products and total synthesis. All products are produced by marine sponges, except 13, 15, 41 (nudibranch) and 65, 66 (ascidian).

#	Product	Species	References	Total synthesis
1	Leucettidine	*Leucetta microraphis*	Cardellina et al. 1981	
2	Naamine A	*Leucetta chagosensis*<br>*Leucetta avocado*	Carmely et al. 1987	
3	Naamine B	*Leucetta chagosensis*	Carmely et al. 1989	
4	Naamine C	*Leucetta chagosensis*	Fu et al. 1997	
5	Naamine D	*Leucetta cf. chagosensis*	Dunbar et al. 2000	
6	5 N,N-Dimethyl naamine D	*Leucetta avocado*	Crews et al. 2003	
7	Naamine E	*Leucetta chagosensis*	Gross et al. 2002	
8	Naamine F	*Leucetta chagosensis*	Hassan et al. 2004	
9	Naamine G	*Leucetta chagosensis*	Hassan et al. 2004	Koswatta & Lovely, 2010a
10	Isonaamine A	*Leucetta chagosensis*	Carmely et al. 1987	Molina et al. 1999; Ermolat'ev et al., 2008
11	Isonaamine B	*Leucetta chagosensis*	Fu et al. 1998	
12	Isonaamine C	*Leucetta chagosensis*	Gross et al. 2002	Nakamura et al. 2003; Ermolat'ev et al. 2008; Lima et al., 2011
13	Dorimidazole A	*Notodoris gardineri*	Alvi et al. 1991	Alvi et al. 1991; Molina et al. 1999
14	Dorimidazole B	*Leucetta chagosensis*	Hassan et al. 2009	Zavesky et al. 2014
15	Preclathridine A	*Notodoris gardineri*	Alvi et al. 1993	Kawasaki et al. 1996; Molina et al. 1999; Koswatta & Lovely, 2009; Zavesky et al. 2014
16	Preclathridine B	*Leucetta chagosensis*	Hassan et al. 2009	Zavesky et al. 2014
17	Leucettamine A	*Leucetta microraphis*	Chan et al. 1993	Boehm et al. 1993
18	Naamidine A	*Leucetta chagosensis*	Carmely et al. 1987, 1989	Aberle et al. 2006; Gibbons et al. 2015
19	Naamidine B	*Leucetta chagosensis*	Carmely et al. 1989	
20	Naamidine C	*Leucetta chagosensis*	Carmely et al. 1989	
21	Naamidine D	*Leucetta chagosensis*	Carmely et al. 1989	
22	Naamidine E	*Leucetta* sp.	Caroll et al. 1993	
23	Naamidine F	*Leucetta* sp.	Caroll et al. 1993	
24	Naamidine G	*Leucetta* sp.	Mancini et al. 1995	Koswatta & Lovely, 2010b
25	Naamidine H	*Leucetta chagosensis*	Tsukamoto et al. 2007	Koswatta & Lovely, 2010a
26	Naamidine I	*Leucetta chagosensis*	Tsukamoto et al. 2007	
27	Pyronaamidine	*Leucetta* sp.	Akee et al. 1990	
28	(2*E*,9*E*)-pyronaamidine 9-(*N*-methylimine)	*Leucetta* sp. cf. *chagosensis*	Plubrukarn et al. 1997	
29	14-hydroxynaamidine A	*Leucetta* sp.	Mancini et al. 1995	
30	14-hydroxynaamidine G	*Leucetta* sp.	Mancini et al. 1995	
31	14-methoxynaamidine A	*Leucetta* sp.	Mancini et al. 1995	
32	14-methoxynaamidine G	*Leucetta* sp.	Mancini et al. 1995	
33	14-oxonaamidine G	*Leucetta* sp.	Mancini et al. 1995	
34	Isonaamidine A	*Leucetta chagosensis*	Carmely et al. 1987, 1989	
35	Isonaamidine B	*Leucetta chagosensis*	Carmely et al. 1989	
36	Isonaamidine C	*Leucetta* sp.	Copp et al. 1998	
37	Isonaamidine D	*Leucetta cf. chagosensis*	Fu et al. 1998	
38	Isonaamidine E	*Leucetta chagosensis*	Gross et al. 2002	Lima et al. 2011
39	Leucettamidine	*Leucetta microraphis*	Chan et al. 1993	
40	Clathidrine A	*Clathrina clathrus*	Ciminiello et al. 1989	Koswatta & Lovely, 2009
41	Clathidrine B	*Notodoris gardineri*	Alvi et al. 1993	
42	Clathidrine C	*Leucetta sp.*	Caroll et al. 1993	
43	Clathridimine	*Clathrina clathrus*	Roué et al. 2010	
44	(9*E*)-Clathridine-9-*N*-(2-sulfoethyl)imine	*Leucetta microraphis*	He et al. 1992	
45	(Clathridine)$_2$Zn	*Clathrina clathrus*	Ciminiello et al. 1989, 1990	
46	(Isonaamidine C)$_2$Zn	*Leucetta* sp.	Alvi et al. 1993	
47	Naamidine A+A	*Leucetta* sp.	Mancini et al. 1995	
48	Naamidine G+G	*Leucetta* sp.	Mancini et al. 1995	
49	Naamidine A+G	*Leucetta* sp.	Mancini et al. 1995	

50	Isonaamidine B+B	*Leucetta* cf. *chagosensis*	Fu et al. 1998	
51	Isonaamidine B+D	*Leucetta* cf. *chagosensis*	Fu et al. 1998	
52	Kealiiquinone	*Leucetta* sp.	Akee et al. 1990	Das et al. 2013
53	2-deoxy-2-aminokealiquinone	*Leucetta chagosensis*	Fu et al. 1997	Das et al. 2013
54	Spirocalcaridine A	*Leucetta* sp.	Edrada et al. 2003	
55	Spirocalcaridine B	*Leucetta* sp.	Edrada et al. 2003	
56	Kealiinine A	*Leucetta chagosensis*	Hassan et al. 2004	Das et al. 2012
57	Kealiinine B	*Leucetta chagosensis*	Hassan et al. 2004	Das et al. 2012; Gibbons et al. 2012
58	Kealiinine C	*Leucetta chagosensis*	Hassan et al. 2004	Das et al. 2012; Gibbons et al. 2012
59	Leucettamine B	*Leucetta microraphis*	Chan et al. 1993	Roué & Bergman, 1999; Chérouvrier et al. 2002; Debdab et al. 2009; Selvaraju & Sun, 2015
60	Leucettamine C	*Leucetta avocado*	Crews et al 2003	
61	Calcaridine A	*Leucetta* sp.	Edrada et al. 2003	Koswatta et al. 2008
62	Spiroleucettadine	*Leucetta* sp.	Ralifo & Crews, 2004; White et al. 2008	Aberle et al. 2007
63	Phorbatopsin A	*Phorbas topsenti*	Nguyen et al. 2012	Ling et al. 2013
64	Phorbatopsin B	*Phorbas topsenti*	Nguyen et al. 2012	
65	Phorbatopsin C	*Phorbas topsenti*	Nguyen et al. 2012	
66	Polyandrocarpamine A	*Polyandrocarpa sp.*	Davis et al. 2002	Davis et al. 2008
67	Polyandrocarpamine B	*Polyandrocarpa sp.*	Davis et al. 2002	Davis et al. 2008
68	Leucosolenamine A	*Leucosolenia* sp.	Ralifo et al. 2007	
69	Leucosolenamine B	*Leucosolenia* sp.	Ralifo et al. 2007	

Supplementary Table S3. Kinase orthologues from unicellular parasites cloned, expressed and tested in this study. *Pf, Plasmodium falciparum; Lm, Leishmania major. Ld, Leishmania donovani; Tb, Trypanosoma brucei; Tc, Trypanosoma cruzi; Cp, Cryptosporidium parvum; Gl, Giardia lamblia; Tc, Toxoplasma gondii.*

Kinase	Parasite	Protein accession number
LmCK1.2	*Leishmania major*	XP_003722496.1
LmDYRK2	*Leishmania major*	XP_001685943.1
LmCLK	*Leishmania major*	XP_001681214.1
LdDYRK3	*Leishmania donovani*	XP_003864768.1
LdDYRK4	*Leishmania donovani*	XP_003860718.1
LdDYRK1B	*Leishmania donovani*	XP_003859543.1
TbCLK1	*Trypanosoma brucei*	XP_829303.1
TcCLK1	*Trypanosoma cruzi*	XP_821361.1
PfGSK-3	*Plasmodium falciparum*	XP_001351197.1
PfCLK1 (PfLAMMER)	*Plasmodium falciparum*	XP_001348605.1
CpLAMMER	*Cryptosporidium parvum*	XP_001388249.1
GlCLK	*Giardia lamblia*	XP_001708093.1
TgCLK	*Toxoplasma gondii*	EEE24592.1

Supplementary Table S4. DiscoveRx KinomeScan® Kinase Selectivity Panel (442 kinases). Enzymes were prepared and interactions assays were run in the presence of 1 µM polyandrocarpamine A, as described in Karaman et al. (2008). A semi-quantitative scoring of this primary screen was estimated. This score relates to a probability of a hit rather than strict affinity. Scores > 10, between 1 - 10 and < 1 indicate the probability of being a false positive is < 20 %, < 10 %, < 5 %, respectively. Scores ≤ 10 are underlined in green.

Kinase	Score
AAK1	100
ABL1(E255K)-phosphorylated	100
ABL1(F317I)-nonphosphorylated	92
ABL1(F317I)-phosphorylated	86
ABL1(F317L)-nonphosphorylated	100
ABL1(F317L)-phosphorylated	98
ABL1(H396P)-nonphosphorylated	95
ABL1(H396P)-phosphorylated	100
ABL1(M351T)-phosphorylated	100
ABL1(Q252H)-nonphosphorylated	100
ABL1(Q252H)-phosphorylated	100
ABL1(T315I)-nonphosphorylated	67
ABL1(T315I)-phosphorylated	85
ABL1(Y253F)-phosphorylated	100
ABL1-nonphosphorylated	90
ABL1-phosphorylated	100
ABL2	87
ACVR1	100
ACVR1B	81
ACVR2A	100
ACVR2B	99
ACVRL1	100
ADCK3	100
ADCK4	98
AKT1	100
AKT2	100
AKT3	100
ALK	100
AMPK-alpha1	100
AMPK-alpha2	87
ANKK1	100
ARK5	71
ASK1	100
ASK2	100
AURKA	100
AURKB	100
AURKC	99
AXL	100
BIKE	70
BLK	100
BMPR1A	100
BMPR1B	42
BMPR2	95
BMX	100
BRAF	100
BRAF(V600E)	96
BRK	100
BRSK1	100
BRSK2	100
BTK	100
CAMK1	100
CAMK1D	100
CAMK1G	100
CAMK2A	98
CAMK2B	90
CAMK2D	100
CAMK2G	99
CAMK4	94
CAMKK1	100
CAMKK2	94
CASK	100
CDC2L1	100
CDC2L2	100
CDC2L5	100
CDK11	100
CDK2	91
CDK3	100
CDK4-cyclinD1	96
CDK4-cyclinD3	98
CDK5	100
CDK7	100
CDK8	100
CDK9	88
CDKL1	100
CDKL2	100
CDKL3	100
CDKL5	100
CHEK1	100
CHEK2	100
CIT	100
CLK1	32
CLK2	45

CLK3	69
CLK4	30
CSF1R	87
CSK	100
CSNK1A1	100
CSNK1A1L	100
CSNK1D	70
CSNK1E	87
CSNK1G1	100
CSNK1G2	100
CSNK1G3	84
CSNK2A1	12
CSNK2A2	26
CTK	100
DAPK1	79
DAPK2	58
DAPK3	50
DCAMKL1	98
DCAMKL2	100
DCAMKL3	94
DDR1	100
DDR2	69
DLK	100
DMPK	100
DMPK2	99
DRAK1	42
DRAK2	51
DYRK1A	3
DYRK1B	25
DYRK2	24
EGFR	99
EGFR(E746-A750del)	100
EGFR(G719C)	95
EGFR(G719S)	100
EGFR(L747-E749del, A750P)	100
EGFR(L747-S752del, P753S)	100
EGFR(L747-T751del,Sins)	92
EGFR(L858R)	93
EGFR(L858R,T790M)	100
EGFR(L861Q)	100
EGFR(S752-I759del)	100
EGFR(T790M)	93
EIF2AK1	100
EPHA1	100
EPHA2	100
EPHA3	87
EPHA4	83
EPHA5	100
EPHA6	100
EPHA7	100
EPHA8	100

EPHB1	89
EPHB2	79
EPHB3	100
EPHB4	100
EPHB6	97
ERBB2	100
ERBB3	100
ERBB4	99
ERK1	100
ERK2	100
ERK3	82
ERK4	100
ERK5	100
ERK8	67
ERN1	100
FAK	94
FER	100
FES	96
FGFR1	100
FGFR2	100
FGFR3	100
FGFR3(G697C)	83
FGFR4	98
FGR	100
FLT1	100
FLT3	100
FLT3(D835H)	94
FLT3(D835Y)	100
FLT3(ITD)	100
FLT3(K663Q)	100
FLT3(N841I)	100
FLT3(R834Q)	100
FLT4	100
FRK	100
FYN	100
GAK	100
GCN2(Kin.Dom.2,S808G)	90
GRK1	100
GRK4	100
GRK7	100
GSK3A	100
GSK3B	100
HCK	100
HIPK1	52
HIPK2	50
HIPK3	19
HIPK4	85
HPK1	100
HUNK	100
ICK	100
IGF1R	100

IKK-alpha	100
IKK-beta	100
IKK-epsilon	89
INSR	90
INSRR	81
IRAK1	28
IRAK3	98
IRAK4	46
ITK	94
JAK1(JH1domain-catalytic)	72
JAK1(JH2domain-pseudokinase)	92
JAK2(JH1domain-catalytic)	97
JAK3(JH1domain-catalytic)	84
JNK1	100
JNK2	100
JNK3	93
KIT	87
KIT(A829P)	91
KIT(D816H)	100
KIT(D816V)	100
KIT(L576P)	99
KIT(V559D)	91
KIT(V559D,T670I)	85
KIT(V559D,V654A)	100
LATS1	100
LATS2	100
LCK	100
LIMK1	100
LIMK2	96
LKB1	100
LOK	100
LRRK2	100
LRRK2(G2019S)	100
LTK	100
LYN	89
LZK	100
MAK	91
MAP3K1	100
MAP3K15	100
MAP3K2	100
MAP3K3	100
MAP3K4	100
MAP4K2	97
MAP4K3	78
MAP4K4	98
MAP4K5	100
MAPKAPK2	100
MAPKAPK5	100
MARK1	100
MARK2	100

MARK3	100
MARK4	100
MAST1	100
MEK1	100
MEK2	100
MEK3	98
MEK4	100
MEK5	78
MEK6	100
MELK	100
MERTK	100
MET	100
MET(M1250T)	97
MET(Y1235D)	100
MINK	100
MKK7	89
MKNK1	63
MKNK2	100
MLCK	100
MLK1	100
MLK2	100
MLK3	100
MRCKA	100
MRCKB	100
MST1	70
MST1R	100
MST2	100
MST3	100
MST4	100
MTOR	84
MUSK	56
MYLK	98
MYLK2	95
MYLK4	96
MYO3A	100
MYO3B	88
NDR1	100
NDR2	91
NEK1	100
NEK11	100
NEK2	100
NEK3	100
NEK4	100
NEK5	95
NEK6	100
NEK7	100
NEK9	100
NIM1	100
NLK	100
OSR1	100
p38-alpha	100

p38-beta	81
p38-delta	100
p38-gamma	100
PAK1	100
PAK2	70
PAK3	100
PAK4	100
PAK6	100
PAK7	79
PCTK1	100
PCTK2	100
PCTK3	100
PDGFRA	100
PDGFRB	94
PDPK1	100
PFCDPK1(P.falciparum)	100
PFPK5(P.falciparum)	100
PFTAIRE2	100
PFTK1	100
PHKG1	100
PHKG2	85
PIK3C2B	100
PIK3C2G	84
PIK3CA	100
PIK3CA(C420R)	100
PIK3CA(E542K)	100
PIK3CA(E545A)	88
PIK3CA(E545K)	100
PIK3CA(H1047L)	100
PIK3CA(H1047Y)	100
PIK3CA(I800L)	99
PIK3CA(M1043I)	93
PIK3CA(Q546K)	81
PIK3CB	100
PIK3CD	100
PIK3CG	100
PIK4CB	100
PIM1	68
PIM2	21
PIM3	38
PIP5K1A	88
PIP5K1C	100
PIP5K2B	100
PIP5K2C	100
PKAC-alpha	100
PKAC-beta	100
PKMYT1	100
PKN1	100
PKN2	100
PKNB(M.tuberculosis)	75
PLK1	96
PLK2	100
PLK3	100
PLK4	100
PRKCD	100
PRKCE	100
PRKCH	77
PRKCI	100
PRKCQ	100
PRKD1	91
PRKD2	92
PRKD3	98
PRKG1	100
PRKG2	93
PRKR	98
PRKX	90
PRP4	100
PYK2	100
QSK	100
RAF1	100
RET	100
RET(M918T)	100
RET(V804L)	100
RET(V804M)	100
RIOK1	100
RIOK2	100
RIOK3	93
RIPK1	100
RIPK2	99
RIPK4	100
RIPK5	83
ROCK1	71
ROCK2	93
ROS1	100
RPS6KA4(Kin.Dom.1-N-terminal)	100
RPS6KA4(Kin.Dom.2-C-terminal)	22
RPS6KA5(Kin.Dom.1-N-terminal)	100
RPS6KA5(Kin.Dom.2-C-terminal)	73
RSK1(Kin.Dom.1-N-terminal)	81
RSK1(Kin.Dom.2-C-terminal)	100
RSK2(Kin.Dom.1-N-terminal)	92
RSK3(Kin.Dom.1-N-terminal)	94
RSK3(Kin.Dom.2-C-terminal)	100
RSK4(Kin.Dom.1-N-terminal)	100
RSK4(Kin.Dom.2-C-terminal)	94
S6K1	100
SBK1	100
SgK110	100

SGK3	100
SIK	100
SIK2	94
SLK	100
SNARK	100
SNRK	100
SRC	100
SRMS	100
SRPK1	100
SRPK2	100
SRPK3	100
STK16	100
STK33	100
STK35	95
STK36	96
STK39	100
SYK	100
TAK1	100
TAOK1	76
TAOK2	100
TAOK3	87
TBK1	100
TEC	98
TESK1	100
TGFBR1	100
TGFBR2	100
TIE1	81
TIE2	98
TLK1	94
TLK2	100

TNIK	100
TNK1	100
TNK2	100
TNNI3K	98
TRKA	95
TRKB	100
TRKC	82
TRPM6	100
TSSK1B	100
TTK	100
TXK	100
TYK2(JH1domain-catalytic)	100
TYK2(JH2domain-pseudokinase)	100
TYRO3	100
ULK1	94
ULK2	100
ULK3	100
VEGFR2	100
VRK2	100
WEE1	100
WEE2	100
YANK1	100
YANK2	94
YANK3	100
YES	100
YSK1	100
YSK4	100
ZAK	95
ZAP70	100

SUPPLEMENTARY FIGURES

Supplementary Figure S1. ***Leucetta microraphis*** (syntype PMJ 133). **A.** Sponge after fixation. **B.** Tangential section of the cortex. **C.** Tangential section of the atrium. **D.** Small triactine. **E.** Colossal triactine. **F.** Small tetractine. **G.** Sponge *in vivo* (not the syntype - photo by A. Padua).

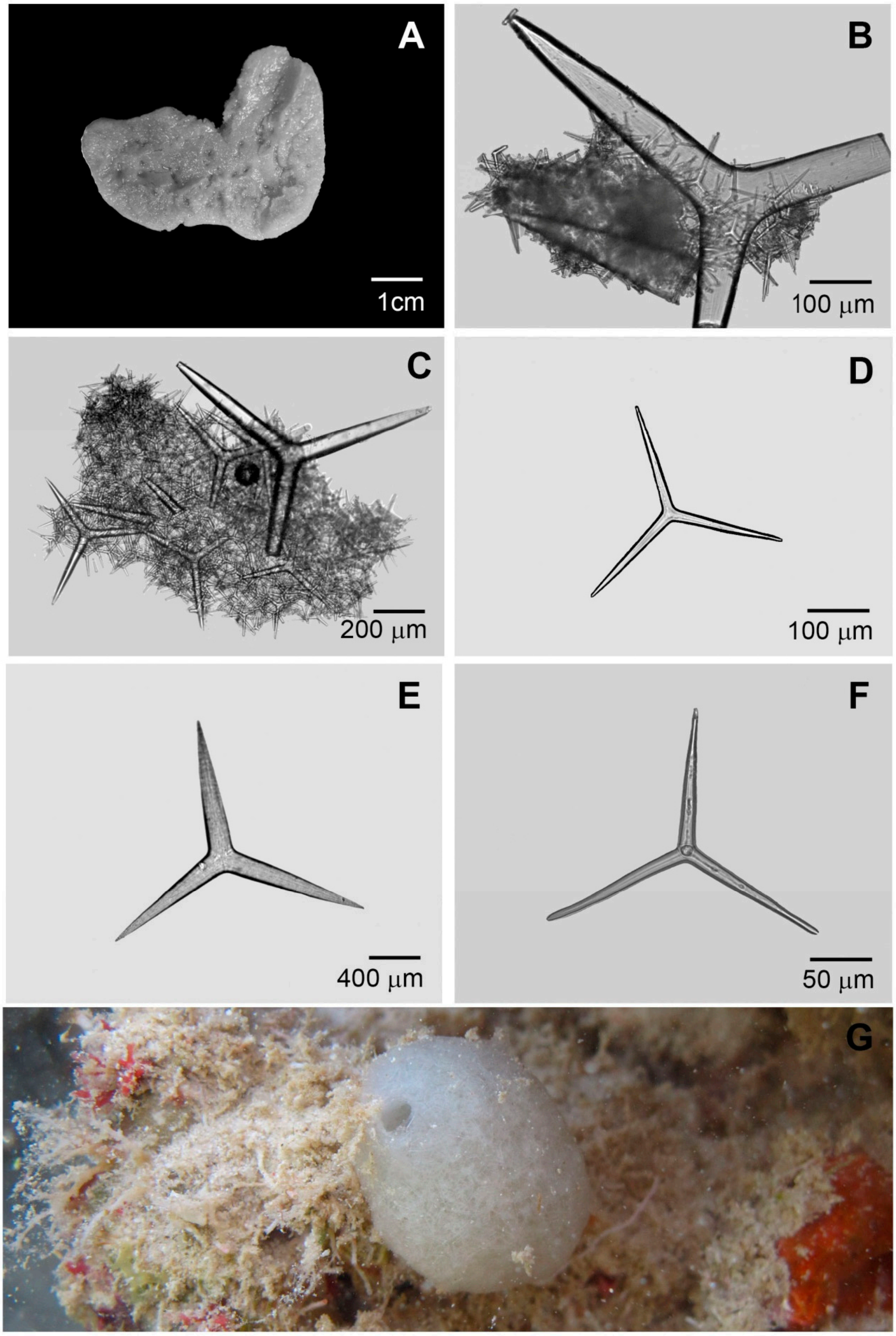

Supplementary Figure S2. *Leucetta chagosensis* (holotype BMNH 1920.12.9.51). **A.** Sponge after fixation. **B.** Tangential section of the cortex. **C.** Tangential section of the atrium. **D.** Small triactine. **E.** Colossal triactine. **F.** Small tetractine. **G.** Sponge *in vivo* (not the holotype - photo by A. Padua)

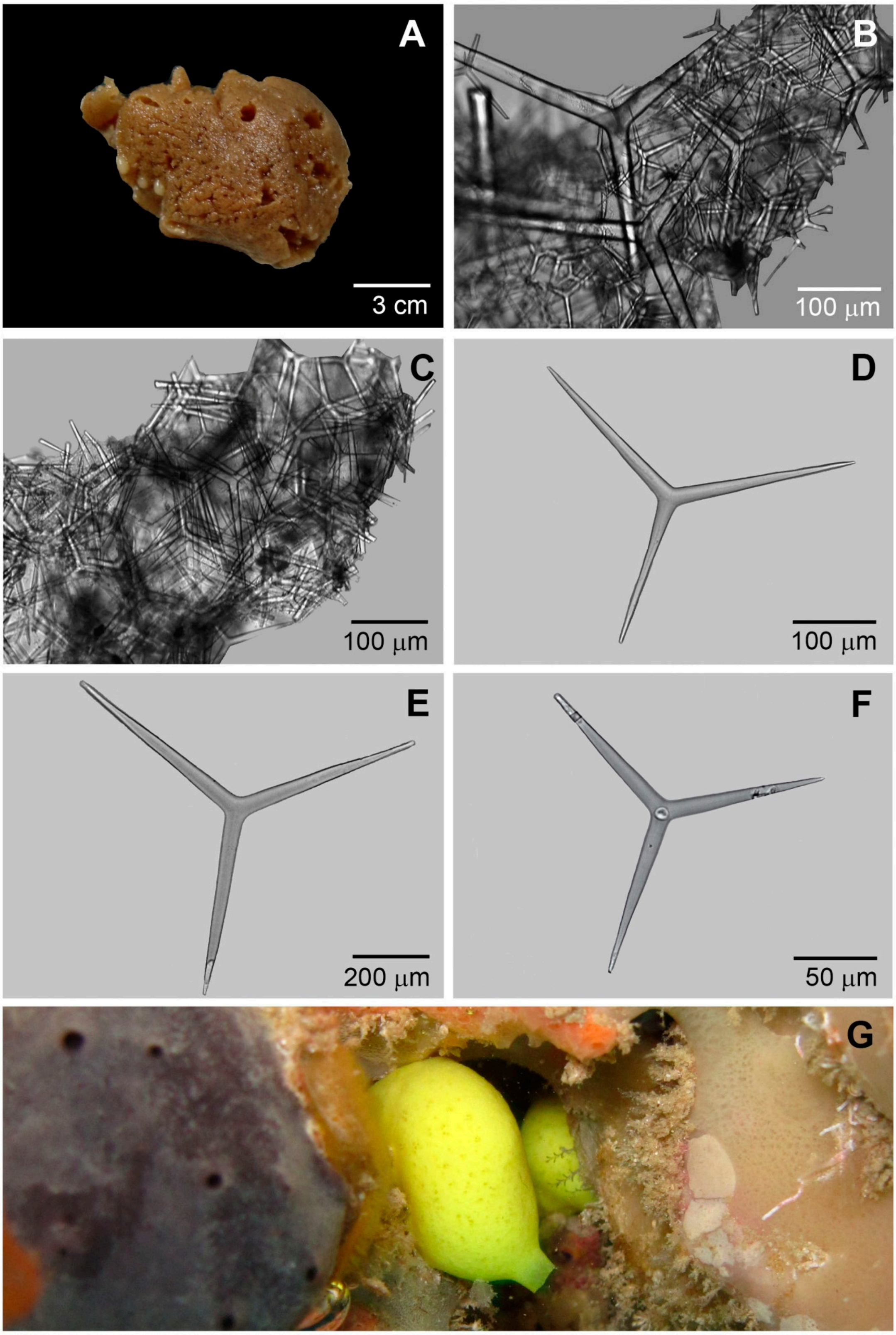

Supplementary Figure S3. *Clathrina clathrus*. **A.** Sponge *in vivo* (photo by J. Vacelet). **B.** Triactine.

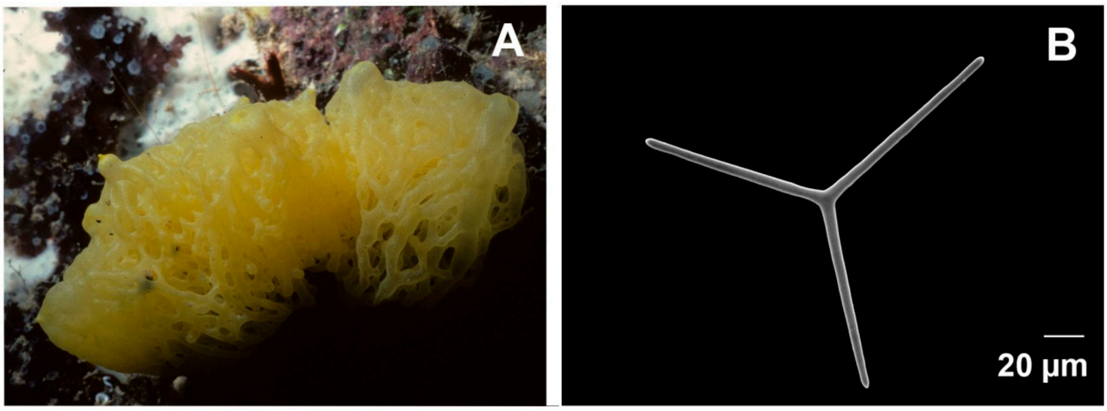

Supplementary Figure S4. Maximum likelihood tree of the nuclear ITS marker of Clathrinida species (subclass Calcinea).

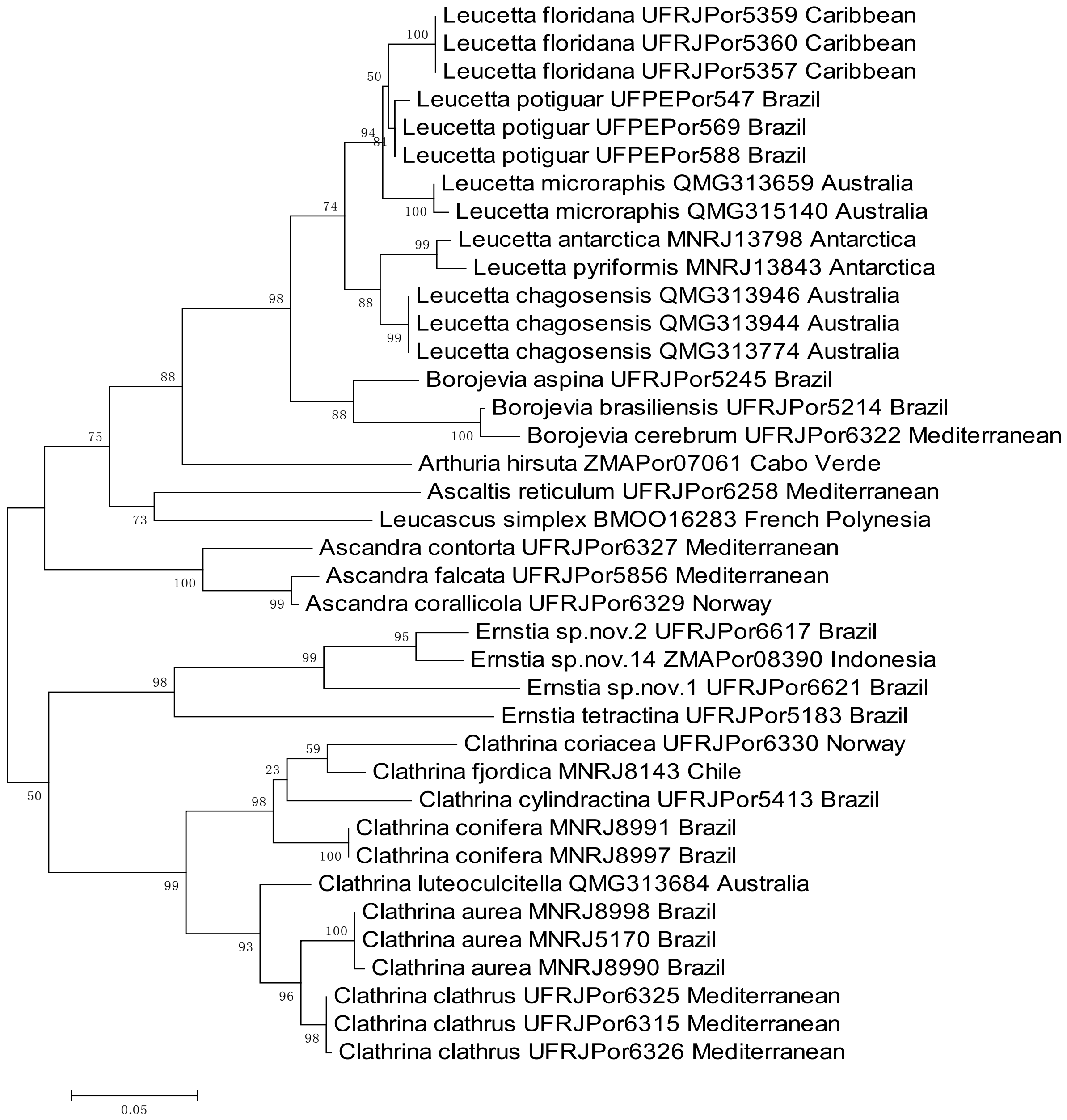

Supplementary Figure S5. Structure of aminoimidazole alkaloids from calcareaous sponges and a few other invertebrates. Organized according to the classification of Roué et al. (2012).

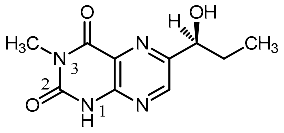

1 Leucettidine

Category I. 2-Aminoimidazole alkaloids

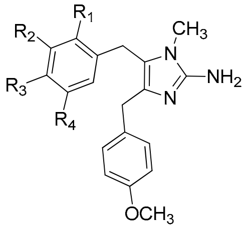

		R_1	R_2	R_3	R_4
**2**	**Naamine A**	H	H	OH	H
**4**	**Naamine C**	OH	OMe	OMe	H
**7**	**Naamine E**	H	OH	OMe	OH
**8**	**Naamine F**	H	OMe	OH	H
**9**	**Naamine G**	H	OMe	OH	OMe

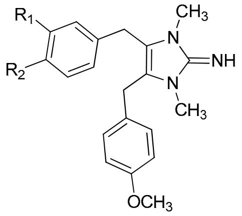

		R_1	R_2
**3**	**Naamine B**	OH	OMe
**6**	**5 *N,N*-Dimethyl naamine D**	H	OMe

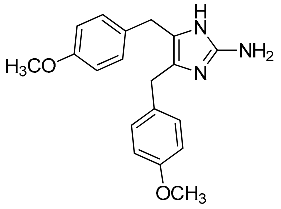

5 Naamine D

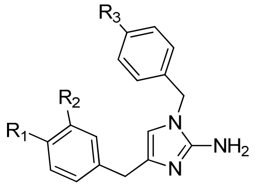

		R_1	R_2	R_3
**10**	**Isonaamine A**	OH	H	OH
**12**	**Isonaamine C**	OMe	OMe	OMe

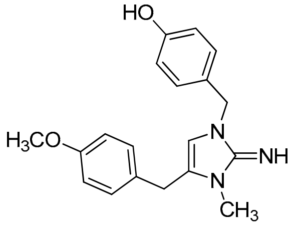

11 Isonaamine B

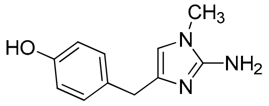

13 Dorimidazole A

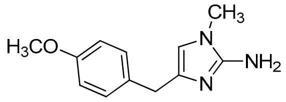

14 Dorimidazole B

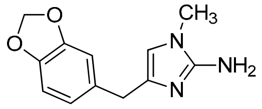

15 PreclatridrineA

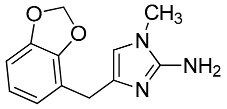

16 Preclathridine B

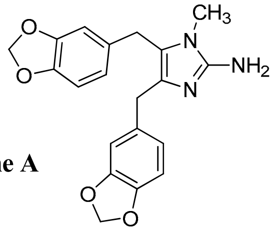

17 Leucettamine A

Category II. 2-Aminoimidazole alkaloids functionalized on the 2-amino group.

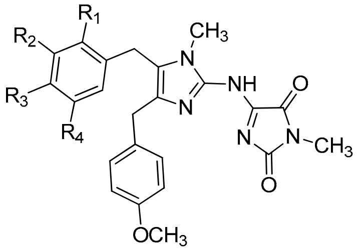

		R_1	R_2	R_3	R_4
**18**	**Naamidine A**	H	H	OH	H
**19**	**Naamidine B**	H	OH	OMe	H
**22**	**Naamidine E**	OH	OMe	OMe	OH
**24**	**Naamidine G**	H	H	OMe	H
**25**	**Naamidine H**	H	OMe	OH	OMe
**27**	**Pyronaamidine**	OH	OMe	OMe	H

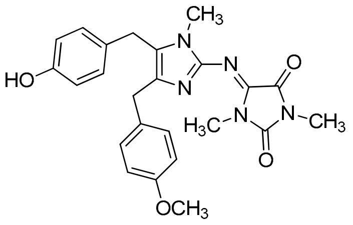

20 Naamidine C

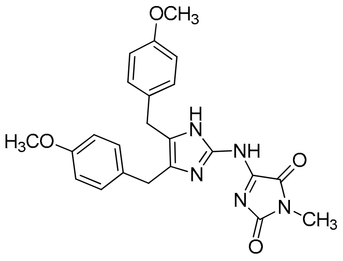

21 Naamidine D

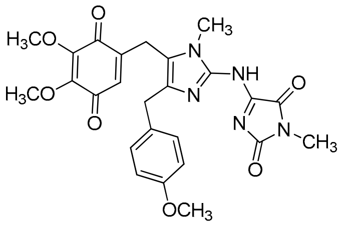

23 Naamidine F

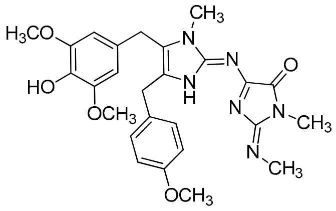

26 Naamidine I

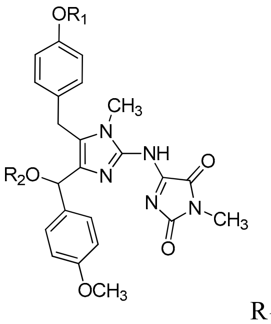

		R_1	R_2
**29**	**14-hydroxynaamidine A**	H	H
**30**	**14-hydroxynaamidine G**	Me	H
**31**	**14-methoxynaamidine A**	H	Me

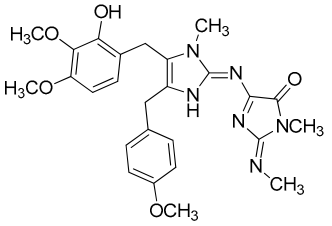

28 (2E,9E)-pyronaamidine 9-(*N*-methylimine)

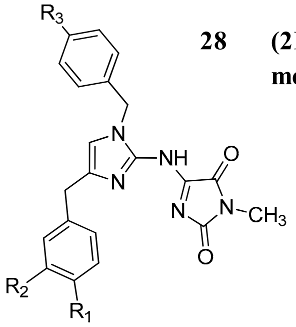

		R_1	R_2	R_3
**34**	**Isonaamidine A**	OH	H	OH
**35**	**Isonaamidine B**	OMe	H	OH
**36**	**Isonaamidine C**	OMe	H	OMe
**38**	**Isonaamidine E**	OMe	OMe	OMe

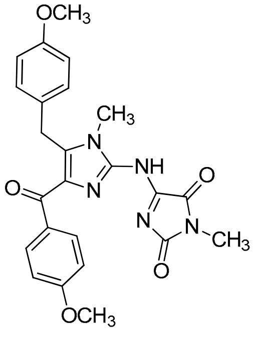

33 14-oxonaamidine G

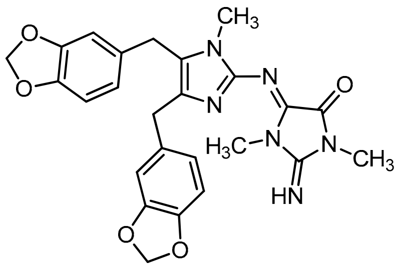

39 Leucettamidine

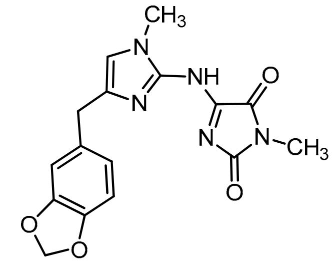

40 Clathridine A

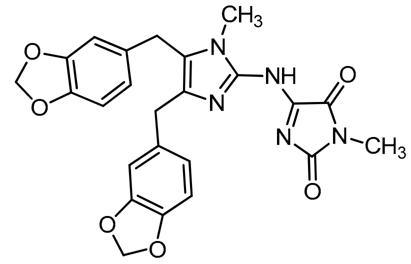

41 Clathridine B

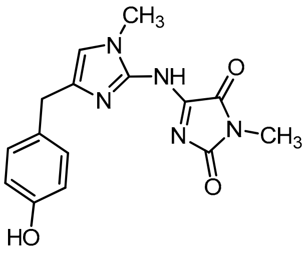

42 Clathridine C

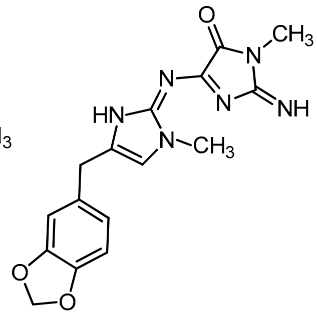

43 Clathridimine

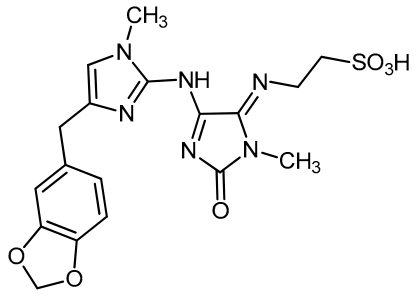

44 (9*E*)-Clathridine 9-*N*-(2-sulfoethyl)imine

Zinc complexes

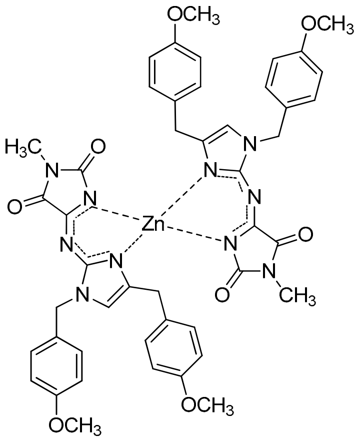

46 (Isonaamidine C)$_2$Zn

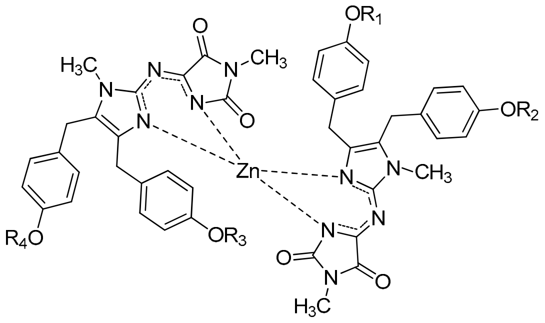

		R_1	R_2	R_3	R_4
**47**	**Naamidine A+A**	Me	H	Me	H
**48**	**Naamidine G+G**	Me	Me	Me	Me
**49**	**Naamidine A+G**	Me	Me	Me	H

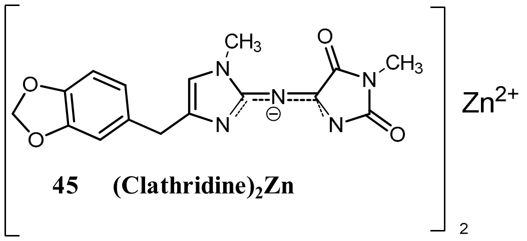

45 (Clathridine)$_2$Zn

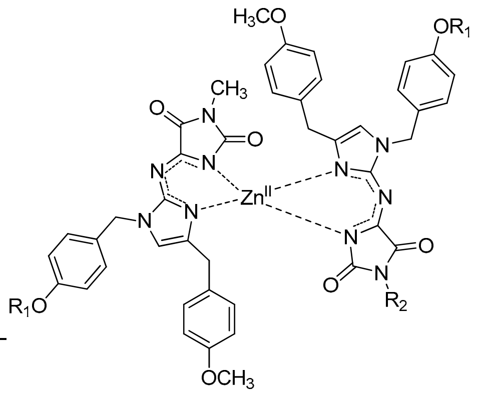

		R_1	R_2
**50**	**Isonaamidine B+B**	H	Me
**51**	**Isonaamidine B+D**	H	H

Category III . 2-Aminoimidazole alkaloids fused in a tetracyclic system.

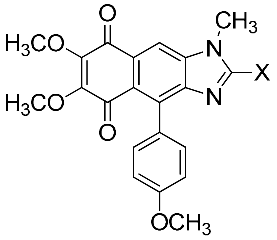

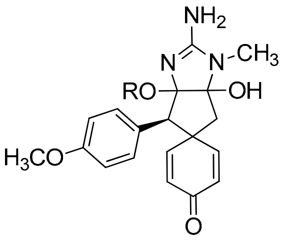

		X
**52**	**Kealiiquinone**	OH
**53**	**2-Deoxy-2-aminokealiquinone**	NH2

		R
**54**	**Spirocalcaridine A**	H
**55**	**Spirocalcaridine B**	Me

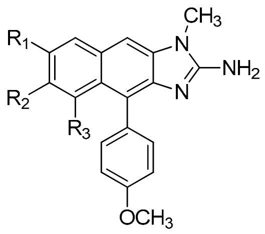

		R_1	R_2	R_3
**56**	**Kealiinine A**	OMe	OH	H
**57**	**Kealiinine B**	OMe	OMe	H
**58**	**Kealiinine C**	OMe	OMe	OMe

Category IV. 2-Aminoimidazolone alkaloids

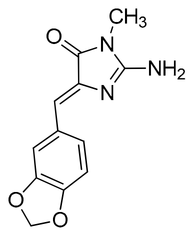

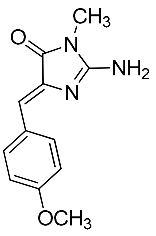

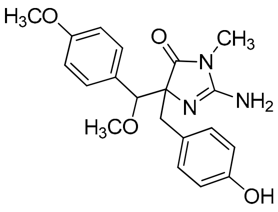

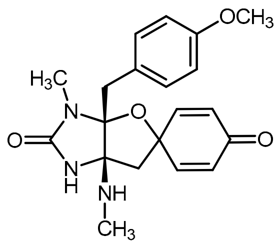

59 **Leucettamine B** **60** **Leucettamine C** **61** **Calcaridine A** **62** **Spiroleucettadine**

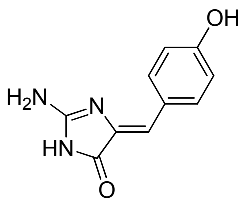

63 **Phorbatopsin A**

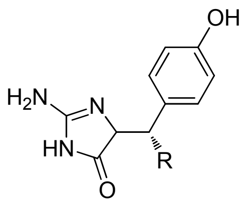

64 **Phorbatopsin B (R = OH)**
65 **Phorbatopsin C (R= H)**

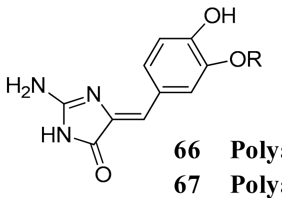

66 **Polyandrocarpamine A (R= Me)**
67 **Polyandrocarpamine B (R= H)**

Other Compounds

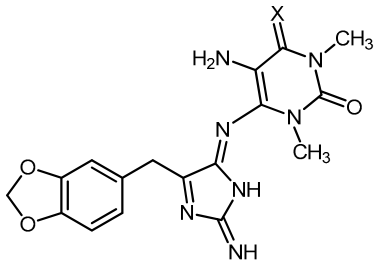

		X
**68**	**Leucosolenamine A**	O
**69**	**Leucosolenamine B**	NMe

SUPPLEMENTARY REFERENCES

Aberle, N. S.; Lessene, G.; Watson, K. G. A concise total synthesis of naamidine A. *Org. Lett.* **2006**, *8*, 419-421.

Aberle, N.; Ovenden, S.P.B.; Lessene, G.; Watson, K.G.; Smith, B.J. Spiroleucettadine: synthetic studies and investigations towards structural revision. *Tetrahedron Lett.* **2007**, *48*, 2199-2203.

Akee, R.K.; Carroll, T.R.; Yoshida, W.Y.; Scheuer, P.J.; Stout, T.J.; Clardy, J. Two imidazole alkaloids from a sponge. *J. Org. Chem.* **1990**, *55*, 1944-1946.

Alvi, K. A.; Crews, P.; Loughhead, D.G. Structures and total synthesis of 2-aminoimidazoles from a *Notodoris* nudibranch. *J. Nat. Prod.* **1991**, *54*, 1509-1515.

Alvi, K.A.; Peters, B.M.; Hunter, L.M.; Crews, P. 2-Aminoimidazoles and their zinc complexes from indo-pacific *Leucetta* sponges and *Notodoris* nudibranchs. *Tetrahedron* **1993**, *49*, 329-336.

Boehm, J.C.; Gleason, J.G.; Pendrak, I.; Sarau, H.M.; Schmidt, D.B.; Foley, J.J.; Kingsbury, W.D. Synthesis and LTB4 receptor antagonist activities of the naturally occurring LTB4 receptor antagonist Leucettamine A and related analogues. *J. Med. Chem.* **1993**, *36*, 3333-3340.

Borojevic, R.; Klautau, M. Calcareous sponges from New Caledonia. *Zoosystema* **2000**, *22*, 187–201.

Borojevic, R.; Boury-Esnault, N.; Manuel, M.; Vacelet, J. Order *Clathrinida* Hartman, 1958. In: Hooper, J.N.A. & van Soest, R.W.M., editors. Systema Porifera: a guide to the classification of sponges. New York: Kluwer Academic/Plenum Publishers. **2002**, pp. 1141-52.

Cardellina, J.H.; Meinwald, J. Leucettidine, a novel pteridine from the calcareous sponge *Leucetta microraphis*. *J. Org. Chem.* **1981**, *46*, 4782-4784.

Carmely, S.; Ilan, M.; Kashman, Y. 2-Aminoimidazole alkaloids from the marine sponge *Leucetta chagosensis. Tetrahedron* **1989**, *45*, 2193-2200.

Carmely, S.; Kashman, Y. Naamines and naamidines, novel imidazole alkaloids from the calcareous sponge *Leucetta chagosensis. Tetrahedron Lett.* **1987**, *28*, 3003-3006.

Carroll, A.R.; Bowden, B.F.; Coll, J.C. New imidazole alkaloids from the *Sponge Leucetta sp*. and the associated predatory nudibranch *Notodoris gardineri*. *Aust. J. Chem.* **1993**, *46*, 1229-1234.

Chan, G. W.; Mong, S.; Hemling, M.E.; Freyer, A.J.; Offen, P.H.; DeBrosse, C.W.; Sarau, H.M.; Westley, J.W. New leukotriene B_4 receptor antagonist: Leucettamine A and related imidazole alkaloids from the marine sponge *Leucetta microraphi*s. *J. Nat. Prod*. **1993**, *56*, 116-121.

Chérouvrier, J.R.; Carreaux, F.; Bazureau, J.P. Microwave-mediated solventless synthesis of new derivatives of marine alkaloid Leucettamine B. T*etrahedron Lett.* **2002**, *43*, 3581-3584.

Ciminiello, P.; Fattorusso, E.; Magno, S.;Mangoni, A. Clathridine and its zinc complex, novel metabolites from the marine sponge *Clathrina clathrus. Tetrahedron* **1989**, *45*, 3873-3878.

Ciminiello, P.; Fattorusso, E.; Mangoni, A.; Di Blasio, B.; Pavone, V. Structure of clathridine zinc complex, a metabolite of the marine sponge *Clathrina clathrus*. *Tetrahedron* **1990**, *46*, 4387-4392.

Copp, B.R.; Fairchild, C.R.; Cornell, L.; Casazza, A.M.; Robinson, S.; Ireland, C.M. Naamidine A is an antagonist of the epidermal growth factor receptor and an *in vivo* active antitumor agent. *J. Med. Chem.* **1998**, *41*, 3909-3911.

Crews, P.; Clark, D.P.; Tenney, K. Variation in the alkaloids among indo-pacific *Leucetta* sponges. *J. Nat. Prod.* **2003**, *66*, 177-182.

Das, J.; Bhan, A.; Mandal, S.S.; Lovely, C.J. Total syntheses and cytotoxicity of kealiiquinone, 2-deoxy-2-aminokealiiquinone and analogs. *Bioorg. Med. Chem. Lett.* **2013**, *23*, 6183–6187.

Das, J.; Koswatta, P.B.; Jones, J.D.; Yousufuddin, M.; Lovely, C.J. Total syntheses of kealiinines A-C. *Org. Lett.* **2012**, *14*, 6210-6213.

Davis, R.A.; Aalbersberg, W.; Meo, S.; Moreira da Rocha, R.; Ireland, C.M. The isolation and synthesis of polyandrocarpamines A and B. Two new 2-aminoimidazolone compounds from the Fijian ascidian, *Polyandrocarpa sp*. *Tetrahedron Lett.* **2002**, *58*, 3263-3269.

Davis, R.A.; Baron, P.S.; Neve, J.E.; Cullinane, C. A microwave-assisted stereoselective synthesis of polyandrocarpamines A and B. *Tetrahedron Lett.* **2008**, *50*, 880-882.

Debdab, M.; Renault, S.; Eid, S.; Lozach, O.; Meijer, L.; Carreaux, F.; Bazureau, J.-P. An efficient method for the preparation of new analogs of leucettamine B under solvent-free microwave irradiation. *Heterocycles* **2009**, *78*, 1191-1203.

Dendy, A. Synopsis of the Australian *Calcarea Heterocoela* with a proposed classification of the group and descriptions of some new genera and species. *Proc. Roy. Soc. Victoria* **1892**, *5*, 69-116.

Dendy, A. The Percy Sladen trust expedition to the Indian Ocean in 1905 (V). I. Report on the calcareous sponges collected by HMS 'Sealark' in the Indian Ocean. *Trans.Linnean Soc. London* **1913**, *16*, 1-29.

Dunbar, D.C.; Rimoldi, J.M.; Clark, A.M.; Kelly, M.; Hamann, M.T. Anti-cryptococcal and nitric oxide synthase inhibitory imidazole alkaloids from the calcareous sponge *Leucetta cf chagosensis. Tetrahedron* **2000**, *56*, 8795-8798.

Edrada, R.A.; Stessman, C.C.; Crews, P. Uniquely modified imidazole alkaloids from a calcareous *Leucetta sponge*. *J. Nat. Prod.* **2003**, *66*, 939-942.

Ermolat'ev, D.S.; Alifanov, V.L.; Rybakov, V.B.; Babaev, E.V.; Van der Eycken, E.V. A concise microwave-assisted synthesis of 2-aminoimidazole marine sponge alkaloids of the isonaamines Series. *Synthesis* **2008**, *13*, 2083-2088.

Felsenstein, J. 1985. Confidence limits on phylogenies: an approach using the bootstrap. *Evolution 39*, 783-791.

Fu, X.; Barnes, J.R.; Do, T.; Schmitz, F.J. New imidazole alkaloids from the sponge *Leucetta chagosensis*. *J. Nat. Prod.* **1997**, *60*, 497-498.

Fu, X.; Schmitz, F.J.; Tanner, R.S.; Kelly-Borges, M. New imidazole alkaloids and zinc complexes from the Micronesian sponge *Leucetta* cf. *chagosensis*. *J. Nat. Prod.* **1998**, *61*, 384-386.

Gibbons, J.B.; Gligorich, K.M.; Welm, B.E.; Looper R.E. Synthesis of the reported structures for kealiinines B and C. *Org. Lett.* **2012**, *14*, 4734-4737.

Gibbons, J.B.; Salvant, J.M.; Vaden, R.M.; Kwon, K.H.; Welm, B.E.; Looper, R.E. Synthesis of Naamidine A and Selective Access to N(2)-Acyl-2-aminoimidazole Analogues. *J. Org. Chem.* **2015**, *80*, 10076-10085.

Gross, H.; Kehraus S.; Konig, G.M.; Woerheide, G.; Wright, A.D. New and biologically active imidazole alkaloids from two sponges of the genus *Leucetta*. *J. Nat. Prod.* **2002**, *65*, 1190-1193.

Haeckel, E. Die kalkschwämme. Eine monographie, Vols 1-3. **1872**, Berlin: Reimer.

Hassan, W.H.B.; Al-Taweel A.M.; Proksch P. Two new imidazole alkaloids from Leucetta chagosensis sponge. *Saudi Pharmaceutical J.* **2009**, *17*, 295– 298.

Hassan, W.; Edrada, R.; Ebel, R.; Wray, V.; Berg, A.; van Soest, R.;, Wiryowidagdo, S.; Proksch, P. New imidazole alkaloids from the indonesian sponge *Leucetta chagosensis. J. Nat. Prod.* **2004**, *67*, 817-822.

He, H.; Faulkner, D. J.; Lee, A. Y.; Clardy, J. A new imidazole alkaloid from the marine sponge *Leucetta microrhaphis*. *J. Org. Chem.* **1992**, *57*, 2176-2178.

Imesek, M.; Plese, B.; Pfannkuchen, M.; Godrijan, J.; Pfannkuchen, D. M.; Klautau, M.; Cetkovic, H. Integrative taxonomy of four *Clathrina* species of the Adriatic Sea, with the first formal description of *Clathrina rubra* Sarà, 1958. *Organisms Diversity & Evolution* **2014**, *14*, 21-29.

Karaman, M.W.; Herrgard, S.; Treiber, D.K.; Gallant, P.; Atteridge, C.E.; Campbell, B.T.; Chan, K.W.; Ciceri, P.; Davis, M.I.; Edeen, P.T.; Faraoni, R.; Floyd, M.; Hunt, J.P.; Lockhart, D.J.; Milanov, Z.V.; Morrison, M.J.; Pallares, G.; Patel, H.K.; Pritchard, S.; Wodicka, L.M.; Zarrinkar, P.P. A quantitative analysis of kinase inhibitor selectivity. *Nat. Biotechnol.* **2008**, *26*, 127-132.

Katoh, S.; Standley, D.M. MAFFT multiple sequence alignment software version 7: improvements in performance and usability (Outlines version 7). *Mol. Biol. Evol.* **2013**, *30*, 772-780.

Kawasaki, I.; Taguchi, N.; Yoneda, Y.; Yamashita, M.; Ohta, S. Highly effective procedure for introduction of amino group into the 2-position of imidazole ring. *Heterocycles* **1996**, *43*, 1375-1379.

Klautau, M.; Azevedo, F.; Cóndor-Luján, B.; Rapp, H.T.; Collins, A.; Russo, C.A. A molecular phylogeny for the order *Clathrinida* rekindles and refines Haeckel's taxonomic proposal for calcareous sponges. *Integr. Comp. Biol.* **2013**, *53*, 447-461.
Klautau, M.; Valentine, C. Revision of the genus *Clathrina* (*Porifera, Calcarea*). *Zool. J. Linnean Soc.* **2003**, *139*, 1-62.
Koswatta, P.B.; Lovely, C.J. Expedient total syntheses of preclathridine A and clathridine A *Tetrahedron Letters* **2009**, *50*, 4998–5000.
Koswatta, P.B.; Lovely, C.J. Concise total synthesis of naamine G and naamidine H. *Chem. Commun.* **2010a**, *46*, 2148-2150.
Koswatta, P.B.; Lovely, C.J. Total Syntheses of naamidine G and 14-methoxynaamidine G. *Tetrahedron Lett.* **2010b**, *51*, 164-166.
Koswatta, P.B.; Sivappa, R.; Dias, H.V.; Lovely, C.J. Total synthesis of (+/-)-calcaridine A and (+/-)-epi-calcaridine A. *Org. Lett.* **2008**, *10*, 5055-5058.
von Lendenfeld, R. A monograph of the Australian sponges. III. The *Calcispongiae*. *Proc. Linnean Soc. New South Wales* **1885**, *9*, 1083-1150.
Lima, H.M.; Garcia-Barboza, B.J.; Khatibi, N.N.; Lovely, C.J. Total syntheses of isonaamine C and isonaamidine E. *Tetrahedron Lett.* **2011**, *52*, 5725-5727.
Ling, Y.; Wang, Z.Q.; Xiao, Y.A.; Zhu, C.; Shen, L.; Wang, X.M.; Hui, Y.; Wang, X.Y. Benzylidene 2-aminoimidazolones derivatives: synthesis and in vitro evaluation of anti-tumor carcinoma activity. *Chem. Pharm. Bull. (Tokyo)*, **2013**, *61*, 1081-1084.
Mancini, I.; Guella, G.; Debitus, C.; Pietra, F. Novel naamidine-type alkaloids and mixed-ligand zinc(II) complexes from a calcareous sponge, *Leucetta* sp., of the Coral Sea. *Helv. Chim. Acta* **1995**, *78*, 1178-1184.
Molina, P.; Fresneda, P.M.; Sanz, M.A. Synthesis of marine alkaloids isonaamine A, dorimidazole A, and preclathridine A. Imminophosphorane-mediated preparation of 2-amino-1,4-disubstituted imidazoles from α-azido esters. *J. Org. Chem* **1999**, *64*, 2540-2544.
Nakamura, S.; Kawasaki, I.; Yamashita, M.; Ohta, S. 1-Methyl-3-trimethylsilylparabanic acid as an effective reagent for the preparation of N-substituted (1-methyl-2,5-dioxo-1,2,5H-imidazolin-4-yl)amines and its application to the total synthesis of isonaamidines A and C, antitumor imidazole alkaloids. *Heterocycles* **2003**, 60, 583-598.
Nguyen, T.D.; Nguyen, X.C.; Longeon, A.; Keryhuel, A.; Le, M.H.; Kim, Y.H.; Chau, V..M.; Bourguet-Kondracki, M.L. Antioxidant benzylidene 2-aminoimidazolones from the Mediterranean sponge *Phorbas topsenti*. *Tetrahedron*, **2012**, *68*, 9256-9259.
Plubrukarn, A.; Smith, D.W.; Cramer, R.E.; Davidson, B.S. (2*E*,9*E*)-pyronaamidine 9-(*N*-methylimine), a new imidazole alkaloid from the Northern Mariana islands sponge *Leucetta* sp. cf. *chagosensis*. *J. Nat. Prod.* **1997**, *60*, 712-715.
Ralifo, P.; Tenney, K.; Valeriote, F.A.; Crews, P. A distinctive structural twist in the aminoimidazole alkaloids from a calcareous marine sponge: isolation and characterization of leucosolenamines A and B. *J. Nat. Prod.* **2007**, *70*, 33-38.
Ralifo, P.; Crews, P. A new structural theme in the imidazole-containing alkaloids from a calcareous *Leucetta* sponge. *J. Org. Chem.* **2004**, *69*, 9025-9029.
Rossi, A.L.; Russo, C.A.M.; Solé-Cava, A.M.; Rapp, H.T.; Klautau, M. Phylogenetic signal in the evolution of body colour and spicule skeleton in calcareous sponges. *Zool. J. Linnean Soc.* **2011**, *163*, 1026-1034.
Roué, M., Domart-Coulon I., Ereskovsky A., Djediat C., Perez T., Bourguet-Kondracki M. Cellular localization of clathridimine, an antimicrobial 2-aminoimidazole alkaloid produced by the Mediterranean calcareous sponge *Clathrina clathrus*. *J. Nat. Prod.* **2010**, *73*, 1277–1282.
Roué, M., Quévrain, E., Domart-Coulon, I., Bourguet-Kondracki, M.L. Assessing calcareous sponges and their associated bacteria for the discovery of new bioactive natural products. *Nat. Prod. Rep.* **2012**, 29, 739-751.

Roué, N.; Bergman, J. Synthesis of the marine alkaloid Leucettamine B. *Tetrahedron* **1999**, *55*, 14729-l4738.

Selvaraju, M.; Sun, C.M. Unprecedented one-pot chemocontrolled entry to thioxoimidazolidinones and aminoimidazolones: synthesis of kinase inhibitor leucettamine B. *ACS Comb. Sci.* **2015**, *17*, 182-189.

Solé-Cava, A.M.; Klautau, M.; Boury-Esnault, N.; Borojevic, R.; Thorpe, J.P. Genetic evidence for cryptic speciation in allopatric populations of 2 cosmopolitan species of the calcareous sponge genus *Clathrina*. *Marine Biol.* **1991**, *111*, 381-386.

Tamura, K.; Stecher, G.; Peterson, D.; Filipski, A.; Kumar, S. MEGA6: Molecular evolutionary genetics analysis (Version 6.0). *Mol. Biol. Evol.* **2013**, *30*, 2725–2729.

Tsukamoto, S.; Kawabata, T.; Kato, H.; Ohta, T.; Rotinsulu, H.; Mangindaan, R.E.P.; van Soest, R.W.M.; Ukai, K.; Kobayashi,H.; Namikoshi, M. Naamidines H and I, cytotoxic imidazole alkaloids from the indonesian marine sponge *Leucetta chagosensis*. *J. Nat. Prod.* **2007**, *70*, 1658-1660.

Valderrama, D.; Rossi, A.L.; Solé-Cava, A.M.; Rapp, H.T. ; Klautau, M. Revalidation of *Leucetta floridana* (Haeckel, 1872) (*Porifera, Calcarea*): a widespread species in the tropical western Atlantic. *Zool. J. Linnean Soc.* **2009**, *157*, 1-16.

White, K.N.; Amagata, T.; Oliver, A.G.; Tenney, K.; Wenzel, P.J.; Crews, P. Structure revision of spiroleucettadine, a sponge alkaloid with a bicyclic core meager in H-atoms. *J. Org. Chem.* **2008**, *73*, 8719-8722.

Wörheide, G.; Epp, S.L.; Macis, L. Deep genetic divergences among Indo-Pacific populations of the coral reef sponge *Leucetta chagosensis* (*Leucettidae*): founder effects, vicariance, or both? *BMC Evol. Biol.* **2008**, *8*, 24.

Wörheide, G., Hooper, J.N.A. *Calcarea* from the Great Barrier Reef. 1: Cryptic *Calcinea* from Heron Island and Wistari Reef (Capricorn-Bunker Group). *Memoirs Queensland Mus.* **1999**, *43*, 859-891.

Wörheide, G.; Hooper, J.N.A., Degnan, B.M. Phylogeography of western Pacific *Leucetta 'chagosensis'* (*Porifera: Calcarea*) from ribosomal DNA sequences: implications for population history and conservation of the Great Barrier Reef World Heritage Area (Australia). *Mol. Ecol.* **2002**, *11*, 1753-1768.

Zavesky, B.P.; Babij, N.R.; Wolfe, J.P. Synthesis of substituted 2-aminoimidazoles via Pd-catalyzed alkyne carboamination reactions. Application to the synthesis of preclathridine natural products. *Org. Lett.* **2014**, *16*, 4952-4955.
